# Nebivolol Inhibits Hepatocellular Carcinoma via RHOQ and Enhances the Efficacy of Lenvatinib

**DOI:** 10.7150/ijbs.127395

**Published:** 2026-03-25

**Authors:** Saihua Zhang, Hechun Lin, Junming Yu, Yunyu Wu, Changbing Wang, Lin Mao, Yizi Jin, Qin Geng, Shuqing Jiang, Xin Shen, Chao Ge, Taoyang Chen, Jinjun Li, Jing Li, Xiuying Xiao, Hong Li

**Affiliations:** 1 State Key Laboratory of Systems Medicine for Cancer, Shanghai Cancer Institute, Renji Hospital, School of Medicine, Shanghai Jiao Tong University, 25/Ln 2200 Xietu Road, Shanghai 200032, China.; 2 Department of Radiation Oncology, Shengli Oilfield Central Hospital, Dongying, Shandong 257000, China.; 3 Department of Pathology, Qi Dong Liver Cancer Institute, Qidong 226220, China.; 4 Department of Oncology, Huashan Hospital Fudan University, 12 Middle Urumqi Road, Shanghai 200000, China.

**Keywords:** Hepatocellular carcinoma, Nebivolol, β_1_-adrenergic receptor, RHOQ, Drug synergism

## Abstract

Hepatocellular carcinoma (HCC) is associated with high mortality due to late diagnosis, recurrence, and limited therapeutic options. Thus, there is an urgent need for novel treatment approaches and new therapeutic targets. Cardiotoxicity resulting from anticancer therapies has become increasingly prominent, leading to a higher risk of cardiovascular diseases among cancer survivors. In our study, through a drug library screening, we identified that the cardiovascular therapeutic agent nebivolol exerts antitumor effects against HCC both *in vivo* and *in vitro*. Mechanistically, nebivolol suppressed HCC progression by downregulating RHOQ independently of β_1_-adrenergic receptors. Furthermore, the combination of nebivolol and lenvatinib synergistically inhibited HCC proliferation both *in vivo* and *in vitro*. This study provides a rationale for repurposing nebivolol as a combination strategy for HCC therapy.

## Introduction

Primary hepatocellular carcinoma (HCC) is one of the most common types of malignant tumors and ranks as the third leading cause of cancer-related mortality worldwide, with a 5-year relative survival rate of approximately 18% 1-3. HCC is a complex disease driven by multiple factors, and surgical resection is the preferred treatment for early-stage hepatocellular carcinoma 4. However, due to its highly covert biological behavior and aggressive progression of HCC, many patients are already in the intermediate or advanced stages at the time of initial diagnosis, thereby missing the opportunity for curative surgical treatment 2. It is estimated that 50%-60% of HCC patients require systemic therapy 5. Although first-line treatments such as sorafenib, lenvatinib, and immunotherapy can moderately extend overall survival, the overall survival benefit remains limited 6-8. While molecularly targeted and immunotherapy-guided precision treatment strategies are theoretically reasonable, clinical reality is often complex, and many patients predicted to benefit do not achieve the expected outcomes. Therefore, there is a pressing need to develop novel effective therapeutic agents or drug combination regimens.

Cardiovascular disease (CVD) and cancer are the leading causes of death in 127 countries and regions. Globally, of every 10 deaths worldwide, 4 people die from cardiovascular disease, and 3 people die from cancer 1. Cardiovascular disease and cancer are not completely independent; rather, they are interconnected through shared risk factors and overlapping pathophysiological mechanisms. The cardiovascular toxicity caused by anticancer treatments is increasingly evident, leading to a higher risk of CVD among cancer survivors 9-12. Therefore, drug repurposing offers a promising strategy to identify already approved or clinically trialed cardiovascular agents that exhibit standalone antitumor effects or synergistic activity when combined with first-line anticancer regimens. The conventional process of drug discovery and development is a complex and expensive process, requiring over a decade and billions of dollars in investment, with approximately 90% of candidate drugs ultimately failing in development 13. Drug repurposing is an ideal method for identifying new indications for existing drugs and expanding the range of anticancer treatment combinations.

Nebivolol, a highly selective β₁-adrenergic receptor antagonist, is approved for the treatment of hypertension and heart failure 14. Unlike other β₁-adrenergic receptor antagonists, nebivolol has been shown to improve oxidative stress and systemic insulin sensitivity by reducing NADPH oxidase activity and enhancing endothelial nitric oxide synthase activity 15. Therefore, nebivolol may be a favorable treatment option for hypertension in patients with impaired glucose and lipid metabolism, and is also more suitable for hypertension treatment in cases of systemic homeostasis disruption caused by anticancer therapy. Beyond its conventional cardiovascular effects, nebivolol exhibits anticancer potential. For instance, Niu M *et al*. found that nebivolol upregulates FBXL2 expression to inhibit EGFR-driven non-small cell lung cancer (NSCLC) growth, and its combination with osimertinib exhibits strong inhibitory effects on osimertinib-resistant non-small cell lung cancer 16. Nebivolol also significantly induces mitochondrial-mediated apoptosis in melanoma 17 and blocks oxidative phosphorylation by synergistically inhibiting the activity of NADH dehydrogenase (complex I) and ATP synthase (complex V), thereby limiting the growth of colorectal and breast cancers 18. Nevertheless, the potential role and underlying mechanisms of nebivolol in HCC remain largely unexplored.

In our study, we identified the antitumor activity of nebivolol against HCC through a drug screening strategy, with validation conducted in both *in vivo* and *in vitro* models. Mechanistically, Nebivolol inhibits the carcinogenesis and metastasis of HCC by downregulating RHOQ expression. Additionally, the combination of nebivolol and lenvatinib demonstrated synergistic anticancer effects in HCC cells by suppressing the activation of EGFR-related signaling pathways. Collectively, these results suggest that the drug repurposing of nebivolol provides a novel therapeutic strategy for the treatment of HCC.

## Methods

### Human cell lines and compounds

The human HCC cell lines PLC/PRF/5, Hep3B, SNU449, and HEK-293T were acquired from the American Type Culture Collection (ATCC, USA). Huh7 cells were obtained from the Riken Cell Bank (Tsukuba, Japan). The MHCC-97H and MHCC-LM3 cells were sourced from the Liver Cancer Institute of Zhongshan Hospital, Fudan University (Shanghai, China). The HCC-LY10 cell line was established from human primary HCC tissues in our laboratory. The mouse cell line Hep53.4 was given by Wang Cun research group. All the prementioned cell lines were cultured in DMEM or PRMI-1640 medium, supplemented with 10% fetal bovine serum (FBS) and 1% penicillin/streptomycin and incubated at 37 °C in a humidified atmosphere with 5% CO_2_.

The FDA-approved Drug library (Catalog No. L1300) was purchased from Selleck Chemicals (Houston, Texas, USA). Nebivolol (Catalog NO. S1645) and Lenvatinib (Catalog NO. S1164) were also purchased from Selleck Chemicals (Houston, Texas, USA).

### Compounds screens

After a 24-hour incubation period, the Huh7, SNU449, and PLC/PRF/5 cell lines (seeded in 96-well plates at a density of 3,000-5,000 cells/per well) were treated with 10 μM solutions of compounds from the FDA-approved drug repository. Cell viability was measured at 24 hours and 48 hours post-treatment initiation using the Cell Counting Kit-8 (CCK-8) assay.

### Colony formation assay

HCC cells, with a seeding density ranging from 3,000 to 7,000 cells per well, were plated in 6-well culture plates. After 24 hours of incubation, drugs were added to the plates, which were then cultured for a duration of 2 days. The plates were subsequently fixed with 10% formalin and stained with crystal violet. HCC cells (MHCC-97H, SNU449, and Huh7) stably transfected with negative control, shRHOQ-1/2, or RHOQ-OE were diluted and incubated in a 6-well plate at a density of 1500 cells/well. After 1-2 weeks, once visible cell clusters had formed, the plates were subsequently fixed with 10% formalin and stained with crystal violet.

### Cell apoptosis assessment

The MHCC-97H, Hep3B, SNU449 and Huh7 cells were incubated and treated with Nebivolol or DMSO for 48 hours. Cell samples were then collected and the Annexin-V/PE apoptosis detection kit (Yeasen, China) along with flow cytometry (BD FACS Celesta, USA) were used to quantify the apoptosis rate of the HCC cells. The experimental procedures were conducted in accordance with the provided protocol.

### Cell cycle detection

The MHCC-97H, Hep3B, SNU449 and Huh7 cells were incubated and treated with Nebivolol or DMSO for 48 hours. The HCC cells were then digested with trypsin and washed by PBS. Subsequently, the cells were fixed with 70% ethanol for 12 hours at 4 °C. After fixation, the cells were washed with PBS and stained with propidium iodide (Sigma-Aldrich, USA). The cell cycle distribution was analyzed by flow cytometry (BD FACS Celesta, USA).

### Wound-healing assay

After undergoing various treatments, the HCC cells were seeded into 6-well plates and allowed to expand until they reached approximately 90% confluence. Straight wounds were created in each well using a pipette tip, and the complete culture medium was replaced with serum-free medium. The wound-healing area was then imaged and measured at 0 hours, 24 hours, and 48 hours post-wounding.

### Transwell assay

After undergoing various treatments, the HCC cells were seeded into the upper chambers of transwell inserts using serum-free medium, while the bottom chambers contained medium supplemented with 10% FBS. Following an incubation period of 10 to 72 hours at 37°C, the chambers were fixed with 10% formalin and subsequently stained with crystal violet. The migrated cells from the upper to the lower chamber were counted in five randomly selected fields.

### RNA extraction and quantitative real-time PCR

Total RNA was isolated from treated HCC cells using TRIzol reagent (Invitrogen, CA, USA), followed by reverse-transcription with the PrimeScript RT kit (Takara, Japan). Relative mRNA levels were determined using TB Green Premix Ex Taq II (Takara, Japan), following to the manufacturers' protocols. Primer sequences are listed in Table S1.

### Western blotting

Proteins were extracted from treated HCC cells using RIPA lysis buffer (Thermo Fisher Scientific, Massachusetts, USA) supplemented with protease and phosphatase inhibitor cocktail (Roche, Basel, Switzerland). The protein samples were then resolved through SDS-PAGE and transferred onto PVDF membranes (Millipore, USA). The membranes were incubated with specific primary antibodies at 4°C overnight. The following day, they were incubated with the corresponding secondary antibodies at room temperature for 2 hours. Specific protein bands were detected using an ECL chemiluminescent substrate (Thermo Fisher Scientific, Massachusetts, USA).

### Mouse tumor models

BALB/c nude mice and wild-type C57BL/6 were purchased from GemPharmatech Co., Ltd (Jiangsu, China). All animal care and experiments were permitted by the Shanghai Cancer Institute Animal Care Committee and the Shanghai Medical Experimental Animal Care Commission, and the study complied with all relevant ethical provisions on animal research. Huh7, MHCC-97H and Hep53.4 cells (2×10^^6^ cells per mouse) were injected subcutaneously into the right posterior flanks of 6-8-week-old female BALB/c nude mice or C57BL/6 mice (n = 5 per group). When the tumor volume reached 100 mm³, the mice were randomly assigned to different groups. Tumor volume was calculated using the modified ellipsoid formula V = ½ length × width^2^, following caliper measurements of the length and width axes of subcutaneous tumors. For hydrodynamic tail vein injection (HTVI), 12.5 μg pT3-myr-AKT-HA (addgene, plasmid #31789), 12.5 μg pT/Caggs-NRASV12 (addgene, plasmid #20205) and 3 μg pCMV(CAT)T7-SB100 (addgene, plasmid #34879) transposase plasmid (or 12.5 μg c-myc-PT3EF1a (addgene, plasmid #92046), 12.5 μg pT2/shp53/GFP4 (addgene, plasmid #20208) and 3 μg SB100 transposase plasmid) were mixed in a sterile saline solution at a volume equivalent to 10% of the body weight of C57BL/6 mice (male, 6-8-week-old, n = 5 per group). The mixture was then injected into the lateral tail vein within 5-7 seconds. After tumor establishment, mice were randomly allocated to receive control, nebivolol (10 mg/kg; 20 mg/kg), lenvatinib (4 mg/kg), or combination therapy. All compounds were administered daily via intraperitoneal injection, with dosage and regimen consistent with monotherapy protocols. Tumor volumes and body weights were measured every other day throughout the treatment period.

### Immunohistochemistry

HCC tissue specimens were obtained from patients undergoing surgical resection at the Qidong Liver Cancer Institute (Qidong, Jiangsu, China) with written informed consent from all participants. A tissue microarray containing 211 HCC samples was subjected to immunohistochemical (IHC) analysis following previously described protocols 19. Briefly, after antigen retrieval with citrate buffer (pH 6.0), tissue sections were incubated overnight at 4 °C with primary antibodies against RHOQ (1: 250) and Ki67 (1: 250) antibodies. The stained sections were examined and imaged using a Carl Zeiss Axioskop 2 microscope (Oberkochen, Germany).

The IHC staining results were evaluated in a double-blinded manner. Two pathologists independently performed semi-quantitative scoring (0-4 points) based on staining intensity and the proportion of positive cells. According to the total score derived from the combined scores, hepatocellular carcinoma patients were categorized into two subgroups: low expression group (score 0-2), and high expression group (score 3-4).

### Synergy determination with SynergyFinder

HCC cells were incubated and treated with varying concentrations of nebivolol and lenvatinib for 48 hours. Cell viability was measured using the CCK-8 assay. The online SynergyFinder software (https://synergyfinder.fimm.fi) was used to calculate drug synergy scoring with the “inhibition index”. Synergy scores greater than 0 were considered indicative of synergism, and scores greater than 10 were considered indicative of strong synergistic effects.

### RNA sequencing and data analysis

Huh7 and SNU449 cells were treated with 8 μM nebivolol or DMSO for 48 hours. Total RNA was then extracted using TRIzol and subjected to mRNA Sequence Analysis (Sinotech Genomics, Shanghai). The total RNA was purified using the Tianmo#TR205-200 kit. RNA quality was assessed using an Agilent Bioanalyzer 2100 (Agilent Technologies, CA, USA), and quantification was performed with a Qubit® 3.0 Fluorometer and NanoDrop One spectrophotometer. Gene Set Enrichment Analysis (GSEA) was applied to identify enriched KEGG pathways and Gene Ontology (GO) terms among differentially expressed genes.

### Statistical analysis

All the results are presented as mean ± standard deviation (SD) and all the *in vitro* experiments were conducted with at least for three replicates. Unless specified otherwise, an unpaired or paired two-tailed student's t-test was used to assess the significance of differences between two groups. Survival curves were generated using the Kaplan-Meier method. The IC_50_ value of nebivolol was determined using the logit method. All data were analyzed using GraphPad Prism 8. * represents *P* < 0.05, which means the differences were statistically significant, ** represents *P* < 0.01, *** represents *P* < 0.001.

## Results

### Nebivolol inhibits the proliferation of HCC cells *in vitro*

**Figure 1:**
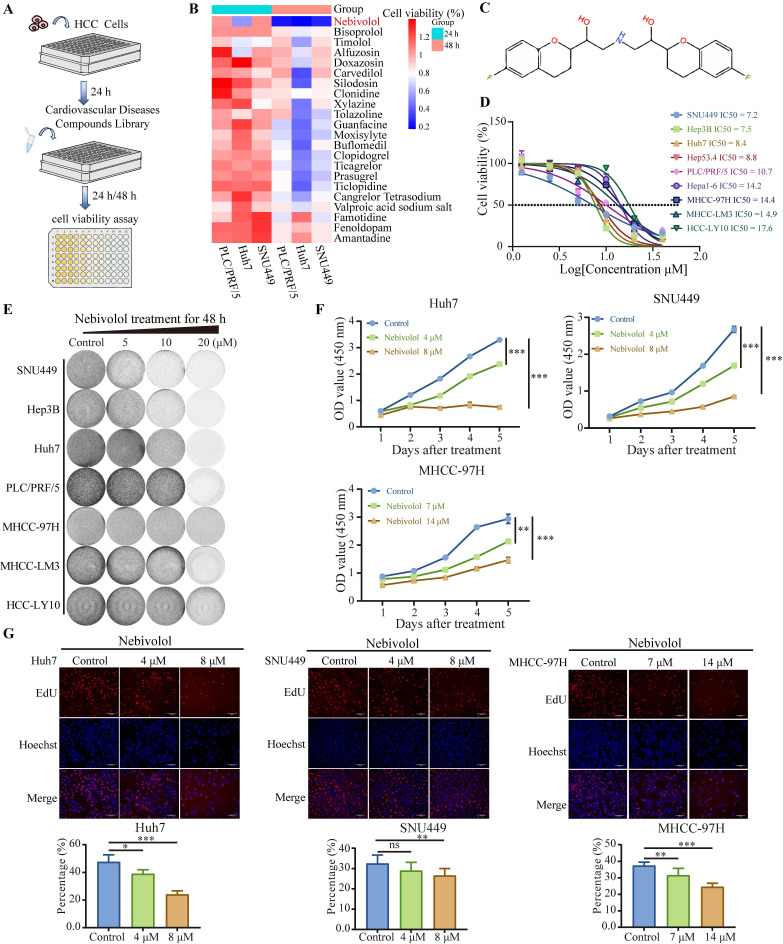
** Nebivolol inhibits the proliferation of HCC cells *in vitro*.** A. Schematic diagram of the drug screening workflow for anti-HCC compounds. B. PLC/PRF/5, Huh 7, and SNU 449 cells were treated by 10 μM FDA-approved drugs for 24 or 48 hours. Viability was quantified using CCK-8 assay. C. Chemical structure of nebivolol. D. Cell viabilities and IC_50_ of HCC cells treated with different concentrations of nebivolol for 48 hours. E. Colony formation assays of HCC cells treated with different concentrations of nebivolol for 48 hours. F. Cell proliferation viability of Huh7, SNU449, and MHCC-97H cells after drug treatment was assessed using the CCK-8 assay. G. Cell proliferation activity in drug-treated Huh7, SNU449, and MHCC-97H cells was evaluated by EdU assay. Scale bar = 100 μm. Data are presented as mean ± SD. * *P* < 0.05, ** *P* < 0.01, *** *P* < 0.001.

To identify potential anti-HCC drugs, we selected 22 FDA-approved small-molecule compounds originally developed for cardiovascular diseases, including those indicated for heart failure, arrhythmia, hypertension, and related disorders. Using a screening concentration of 10 μM, we evaluated the effects of these compounds on the viability of three human HCC cell lines—Huh7, SNU449, and PLC/PRF/5—after 24 and 48 hours of treatment (Fig. 1A). Among them, nebivolol exhibited significant inhibitory effects across all three HCC cell lines (Fig. 1B). The chemical structure of nebivolol is shown in Fig. 1C. We further expanded the set of HCC cell lines and treated them with different concentrations of nebivolol for 48 hours. Both CCK-8 and colony formation assays indicated that nebivolol suppressed HCC cell proliferation in a dose-dependent manner (Fig. 1D and E). Sensitivity to nebivolol varied among the cell lines: SNU449, Hep3B, and Huh7 cells were the most sensitive, with IC₅₀ values of 7.2 µM, 7.5 µM, and 8.4 µM, respectively; whereas HCC-LY10 cells exhibited the lowest sensitivity, with an IC₅₀ of 17.6 µM. Additional proliferation assays using both IC₅₀ and half-IC₅₀ concentrations confirmed that the inhibitory effect strengthened with increasing dose and prolonged exposure time (Fig. 1F and Fig. S1A). Furthermore, EdU cell proliferation assays confirmed that nebivolol inhibited the proliferation activity of HCC cells (Fig. 1G).

Taken together, these results indicate that nebivolol effectively inhibits HCC cell proliferation *in vitro*.

### Nebivolol induces cell cycle arrest and apoptosis while suppressing migration and invasion in HCC cells

**Figure 2:**
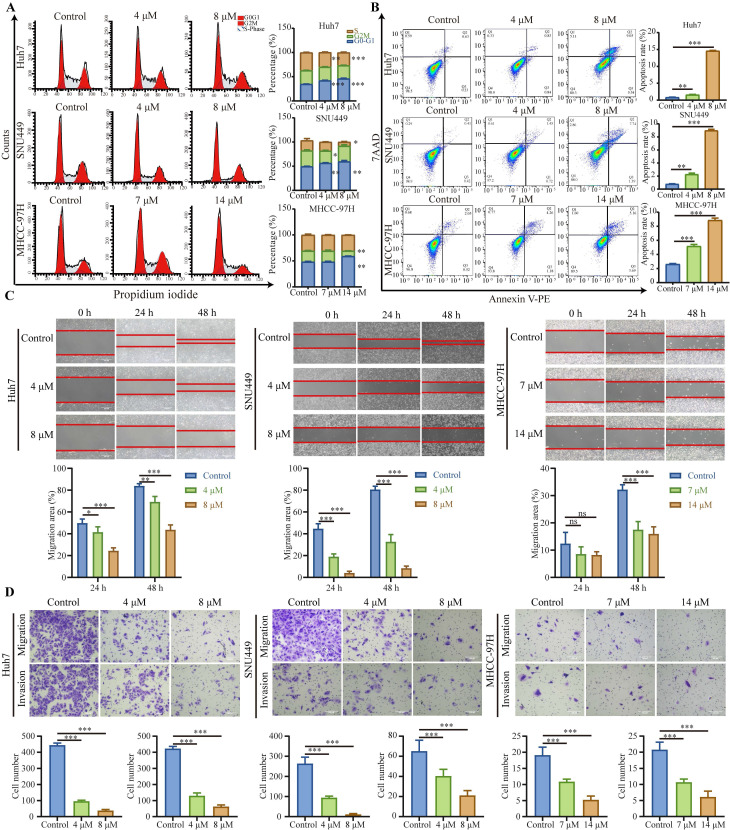
** Nebivolol induces cell cycle arrest and apoptosis while suppressing migration and invasion in HCC cells.** A. Representative images and quantitative analysis of cell cycle distribution in HCC cells treated with different concentrations of nebivolol for 48 hours. B. Representative images and quantitative analysis of apoptosis in HCC cells treated with different concentrations of nebivolol for 48 hours. C. Representative images and quantitative analysis of wound healing assays in HCC cells treated with different concentrations of nebivolol for 24 and 48 hours. Scale bar = 200 μm. D. Representative images and quantitative analysis of transwell migration and invasion images in HCC cells treated with different concentrations of nebivolol. Scale bar = 100 μm. Data are presented as mean ± SD. * *P* < 0.05, ** *P* < 0.01, *** *P* < 0.001.

As demonstrated above, nebivolol effectively inhibits HCC cell proliferation. Given that abnormal proliferation of tumor cells is closely associated with abnormalities in the cell cycle 20, we further examined whether nebivolol influences cell cycle progression in HCC cells. Flow cytometry analysis revealed that nebivolol treatment induced significant cell cycle arrest in HCC cells, characterized by a dose-dependent increase in the proportion of cells in the G0/G1 phase and a concurrent decrease in S-phase cells (Fig. 2A and Fig. S1B). This result is consistent with previous studies in vascular smooth muscle cells, which also reported a G1 phase increase upon nebivolol treatment 21. Furthermore, flow cytometry analysis of annexin V/PI staining showed that the apoptosis level of HCC cells significantly increased after 48 hours of nebivolol treatment (Fig. 2B and Fig. S1C).

We further evaluated the effect of nebivolol on metastatic behaviors using wound healing and Transwell assays. The results showed that nebivolol significantly inhibited the migratory and invasive abilities of HCC cells. (Fig. 2C and D). Taken together, these findings demonstrate that nebivolol potently suppresses HCC progression by inducing cell cycle arrest, promoting apoptosis, and inhibiting migration and invasion.

### Nebivolol potently inhibits the growth of HCC tumors *in vivo*

**Figure 3:**
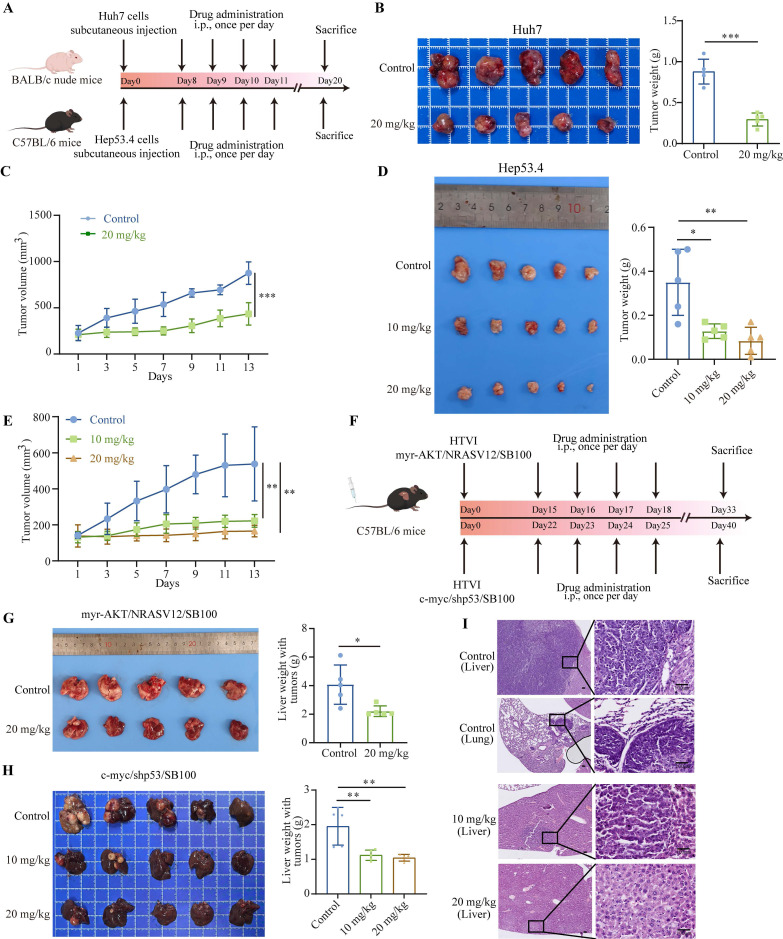
** Nebivolol potently inhibits the growth of HCC tumors *in vivo*.** A. Schematic diagram of the subcutaneous xenograft mouse model and drug administration regimen. B, C The subcutaneous tumor weights (B) and volumes (C) of Huh7 (n = 5) in nude mice treated with control (DMSO) or nebivolol (20 mg/kg) by peritoneal injection. D, E The subcutaneous tumor weights (D) and volumes (E) of Hep53.4 (n = 5) in C57BL/6 mice treated with control (DMSO) or nebivolol (10 mg/kg; 20 mg/kg) by peritoneal injection. F. Schematic of hydrodynamic tail vein injection mouse model and drug administration regimen. G. Tumor-bearing liver images and liver weights from mice treated with myr-AKT/NRASV12/SB100 plasmid combination (n= 5) via intraperitoneal injection of control (DMSO) or nebivolol (20 mg/kg). H. Tumor-bearing liver images and liver weights from mice treated with c-myc/shp53/SB100 plasmid combination (n = 5) via intraperitoneal injection of control (DMSO) or nebivolol (10 mg/kg; 20 mg/kg). I. Representative images of H&E-stained liver tumors induced by c-myc/shp53/SB100 plasmids and H&E-stained lung sections with metastatic nodules. Scale bar = 100 μm. Data are presented as mean ± SD. * *P* < 0.05, ** *P* < 0.01, *** *P* < 0.001.

To further investigate the inhibitory effect of nebivolol on HCC proliferation *in vivo*, we established subcutaneous xenograft models by implanting Huh7 and Hep53.4 cells into mice (Fig. 3A). When the tumor volume reached 100 mm³, the mice were randomly assigned to treatment groups and received daily intraperitoneal injections of either nebivolol or vehicle control (DMSO). Tumor volume and mouse body weight were measured every two days throughout the treatment period. The results showed that nebivolol treatment significantly slowed subcutaneous tumor growth and reduced tumor weight compared to the control group (Fig. 3B-E). Moreover, no significant differences were observed in body weight, liver-to-body weight ratio, or lung-to-body weight ratio between the two groups (Fig. S2B and D). H&E staining of lung tissue also showed no histopathological damage between the two groups (Fig. S2C). The above data indicate that nebivolol has no significant adverse effect on mice.

Using hydrodynamic tail vein injection, we generated two mouse models of spontaneous HCC via delivery of plasmids encoding either myr-AKT/NRASV12/SB100 or c-myc/shp53/SB100 (Fig. 3F). The antitumor effects of nebivolol were further evaluated in these models. Results showed that nebivolol treatment significantly reduced both the size of tumor-bearing livers and liver weight compared to the control group (Fig. 3G and H). Histological examination via H&E staining confirmed that nebivolol administration markedly suppressed liver cancer development (Fig. 3I and Fig. S2A). Furthermore, throughout the treatment, no significant loss in body weight was observed, and lung tissue morphology remained normal, further supporting the safety profile of nebivolol (Fig. S2B and C). Immunohistochemical staining of both subcutaneous and primary liver cancer tissues demonstrated a significant reduction of cell proliferation marker Ki67 in the nebivolol-treated group (Fig. S3A). Notably, in the c-myc/shp53/SB100 model, lung metastases were observed in the control group (3/5), whereas no metastases were observed in the nebivolol-treated group, suggesting that nebivolol may inhibit lung metastasis of liver cancer (Fig. 3I).

Collectively, these results from diverse mouse models demonstrate that nebivolol effectively inhibits the progression and metastasis of HCC *in vivo*.

### Nebivolol downregulates RHOQ expression in HCC cells

**Figure 4:**
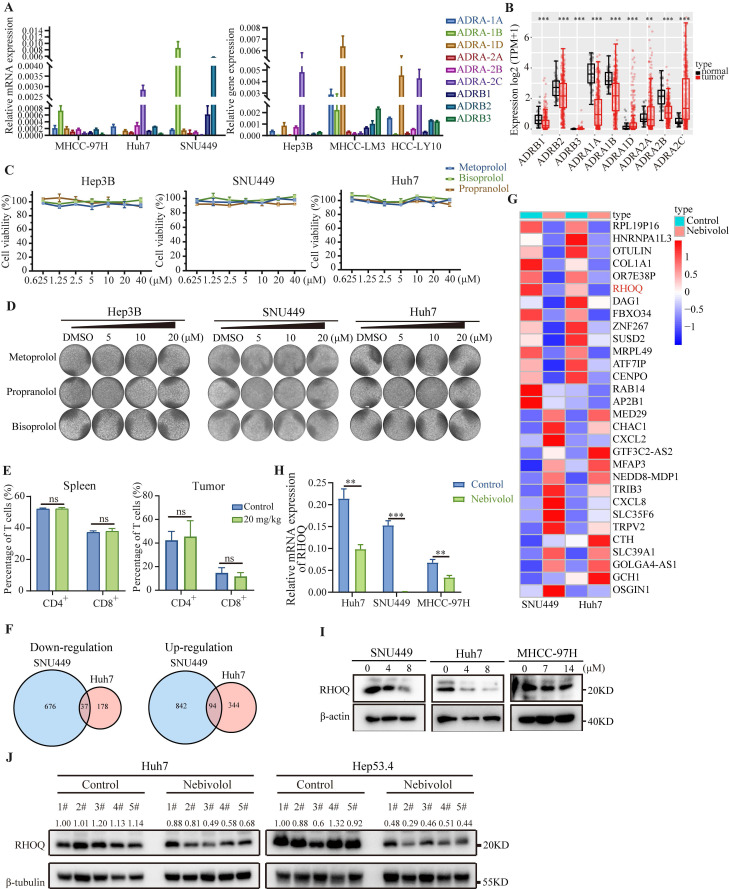
** Nebivolol downregulates RHOQ expression in HCC cells.** A. Adrenergic receptor mRNA expression levels in HCC cell lines by qRT-PCR. B. The mRNA expression of adrenergic receptors between HCC and para-cancerous tissues in TCGA database. C. Cell viability of Huh7, SNU449, and Hep3B cells after 48 hours treatment with metoprolol, propranolol, and bisoprolol. D. Representative images of colony formation. E. Proportions of CD4^+^ and CD8^+^ T cells infiltrating spleens and Hep53.4 subcutaneous tumors in C57BL/6 mice treated with control or nebivolol via intraperitoneal injection. F. The Venn diagram of downregulated and upregulated genes in Huh7 and SNU449 cells after nebivolol treatment compared to control. G. Heatmap of top 15 downregulated and upregulated genes in RNA-sequencing analysis. H. The mRNA expression of RHOQ in HCC cell lines treated with control or nebivolol determined by qRT-PCR. I. Western blotting results of RHOQ expression in HCC cell lines treated with control or nebivolol. J. Western blotting results of RHOQ expression in the subcutaneous tumor (n = 5) treated with control (DMSO) or nebivolol (20 mg/kg). Data are presented as mean ± SD. * *P* < 0.05, ** *P* < 0.01, *** *P* < 0.001.

Numerous studies have reported that adrenergic receptors are highly expressed in various malignant tumors, and their antagonists can inhibit tumor development and progression 22. For example, inhibition of β_2_ adrenergic receptor disrupts PKA signaling, thereby slowing the progression of NSCLC and extending mouse survival 23. In HCC, β_2_-adrenergic receptor signaling plays a crucial role in sustaining cell proliferation and survival and has been linked to acquired resistance to sorafenib 24. Nebivolol is a long-acting and highly selective β_1_ adrenergic receptor antagonist. We therefore first investigated whether its anti-HCC effects are mediated through β_1_-adrenergic receptor blockade. qRT-PCR analysis of adrenergic receptors expression in HCC cell lines revealed extremely low levels of β_1_ adrenergic receptor (Fig. 4A). Analysis of the TCGA database further showed that β_1_-adrenergic receptor expression was significantly lower in HCC tissues compared to non-tumor tissues, and markedly lower compared to other adrenergic receptor subtypes (Fig. 4B). We next treated HCC cells with other β1-adrenergic receptor antagonists—metoprolol, propranolol, and bisoprolol—and assessed their effects on cell proliferation. In contrast to nebivolol, none of these agents significantly inhibited HCC cell growth in CCK-8 or colony formation assays (Fig. 4C and D).

Previous studies have indicated that β_1_-adrenergic receptors function as immune checkpoints regulated by the sympathetic nervous system, modulating T cell activity and reshaping the tumor immune microenvironment to exert anticancer effects 25-28. We therefore performed flow cytometry analysis of T cells isolated from subcutaneous xenograft tumors and spleens of mice bearing Hep53.4 cell-derived tumors. The results showed no significant difference in the proportion of T lymphocytes between the nebivolol-treated and control groups (Fig. 4E and Fig. S4A), suggesting that nebivolol does not inhibit tumor growth by enhancing T cell infiltration. Taken together, these results suggest that nebivolol likely exerts its anti-HCC activity via β_1_-adrenergic receptor-independent mechanisms.

To further investigate the mechanisms of nebivolol's anti-HCC activity, we conducted RNA sequencing on Huh7 and SNU449 cells treated with nebivolol or control. Comparative analysis revealed 131 differentially expressed genes (|fold change| > 1.5), with 37 genes were downregulated and 94 genes upregulated in the nebivolol-treated group (Fig. 4F, Supplementary Table 2). Hallmark pathway enrichment analysis showed significant upregulation of apoptosis pathways, while whereas pathways associated with E2F target genes, mitotic spindle, and DNA replication were significantly down-regulated (Fig. S5A). These results are consistent with our earlier observations that nebivolol promotes apoptosis and inhibits proliferation in HCC cells. Among the differentially expressed genes, RHOQ was one of the most significantly downregulated genes (Fig. 4G). We validated this finding using qRT-PCR and Western blotting, which confirmed that nebivolol treatment reduced RHOQ expression in HCC cells in a dose-dependent manner (Fig. 4H, I). Concordantly, RHOQ expression was also reduced in the subcutaneous tumor from nebivolol-treated mice compared with controls (Fig. 4J). These data suggest that nebivolol may inhibit HCC progression by down regulating RHOQ expression.

### RHOQ is highly expressed in human HCC tissues and predicts poor prognosis

**Figure 5:**
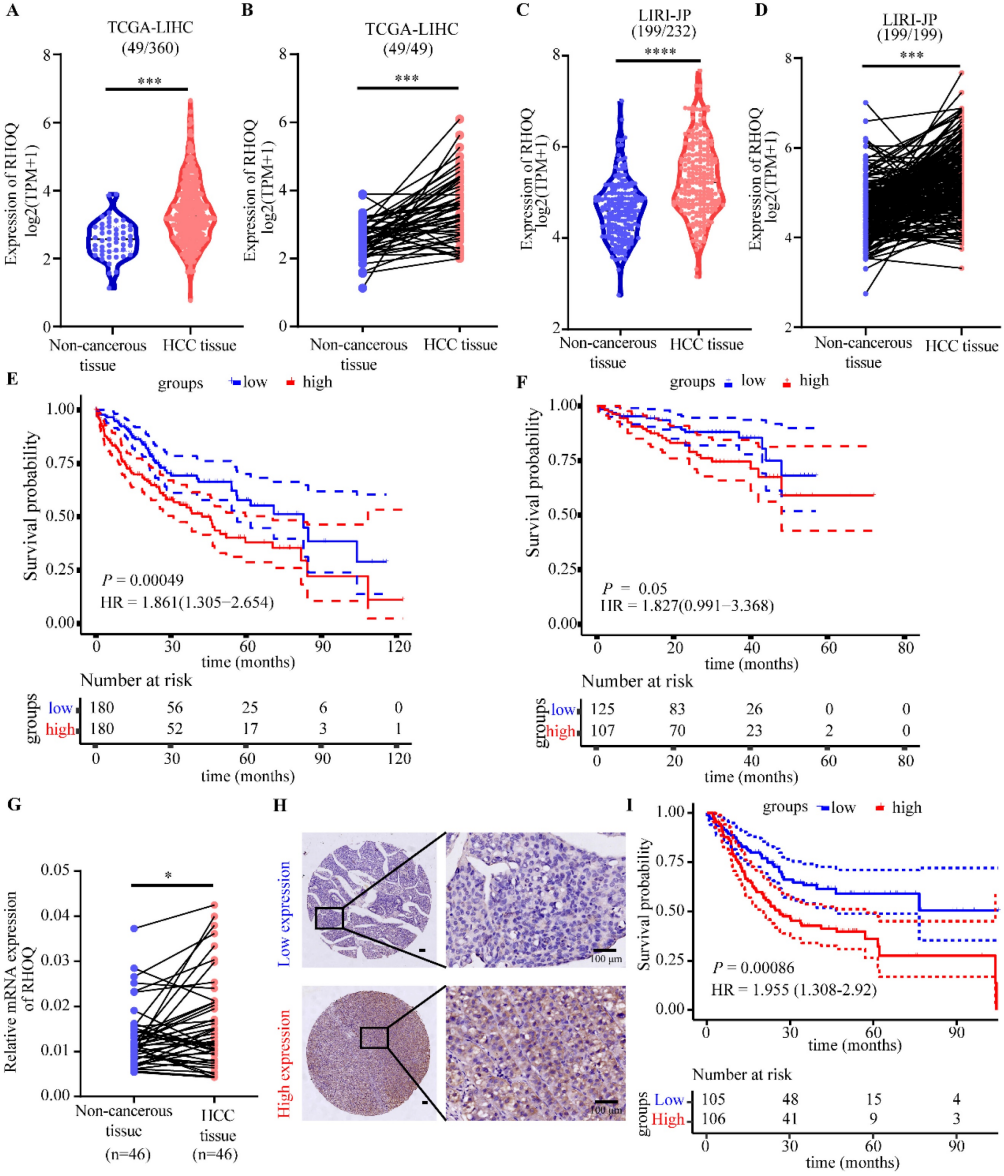
** RHOQ is highly expressed in human HCC tissues and predicts poor prognosis.** A. The mRNA expression of RHOQ in 360 HCC and 49 para-cancerous tissues in TCGA database. B. The mRNA expression of RHOQ in 49 paired HCC and para-cancerous tissues in TCGA database. C. The mRNA expression of RHOQ in 232 HCC and 199 para-cancerous tissues in LIRI-JP database. D. The mRNA expression of RHOQ in 199 paired HCC and para-cancerous tissues in LIRI-JP database. E. Overall survival analysis of HCC patients in the TCGA cohort stratified by the RHOQ expression Kaplan-Meier. HR, Hazard Ratio. F. Kaplan-Meier analysis shows the association of RHOQ mRNA abundance with overall survival in 232 patients with HCC stratified by mean cut-off value of RHOQ. HR, Hazard Ratio. G. RHOQ mRNA levels were detected by qRT-PCR in paired HCC and adjacent non-tumor tissues (n = 46 pairs) from our laboratory. H. Representative images of IHC staining for RHOQ expression in 211 HCC tissue samples from our laboratory. Based on H&E staining scores, the IHC staining of tumor tissues was classified into a low expression group (n = 105) and a high expression group (n = 106). Scale bar = 100 μm. I. Kaplan-Meier analysis of overall survival in our laboratory's HCC cohort stratified by RHOQ expression. HR, Hazard Ratio. Data are presented as mean ± SD. * *P* < 0.05, ** *P* < 0.01, *** *P* < 0.001.

By analyzing HCC data from the TCGA and LIRI-JP database, we found that RHOQ expression levels were significantly higher in HCC tissues than in adjacent normal tissues (Fig. 5A - D). Kaplan-Meier survival analysis indicated that patients with high RHOQ expression had significantly shorter overall survival (Fig. 5E and F). qRT-PCR analysis of 46 paired clinical samples from our laboratory confirmed that RHOQ expression was significantly elevated in HCC tissues compared to adjacent normal tissue (Fig. 5G). Further immunohistochemical analysis of a tissue microarray comprising 211 HCC patients' samples revealed that 106 samples (50.2%) exhibited high RHOQ protein expression, while 105 samples (49.8%) showed low expression (Fig. 5H). Kaplan-Meier survival analysis showed that high RHOQ expression was significantly associated with reduced overall survival, consistent with TCGA and LIRI-JP databases (Fig. 5I). Survival analysis of multiple cancers in the TCGA database revealed that patients with high RHOQ expression had significantly shorter overall survival than those with lower expression in low-grade glioma, cervical squamous cell carcinoma, colorectal cancer, and hepatocellular carcinoma (Fig. S6A).

These results collectively indicate that RHOQ is frequently overexpressed in HCC and correlates with unfavorable clinical outcomes, suggesting its potential utility as a prognostic biomarker and highlighting its promise as a therapeutic target for HCC.

### RHOQ promotes HCC cells' proliferation and modulates sensitivity to nebivolol

**Figure 6:**
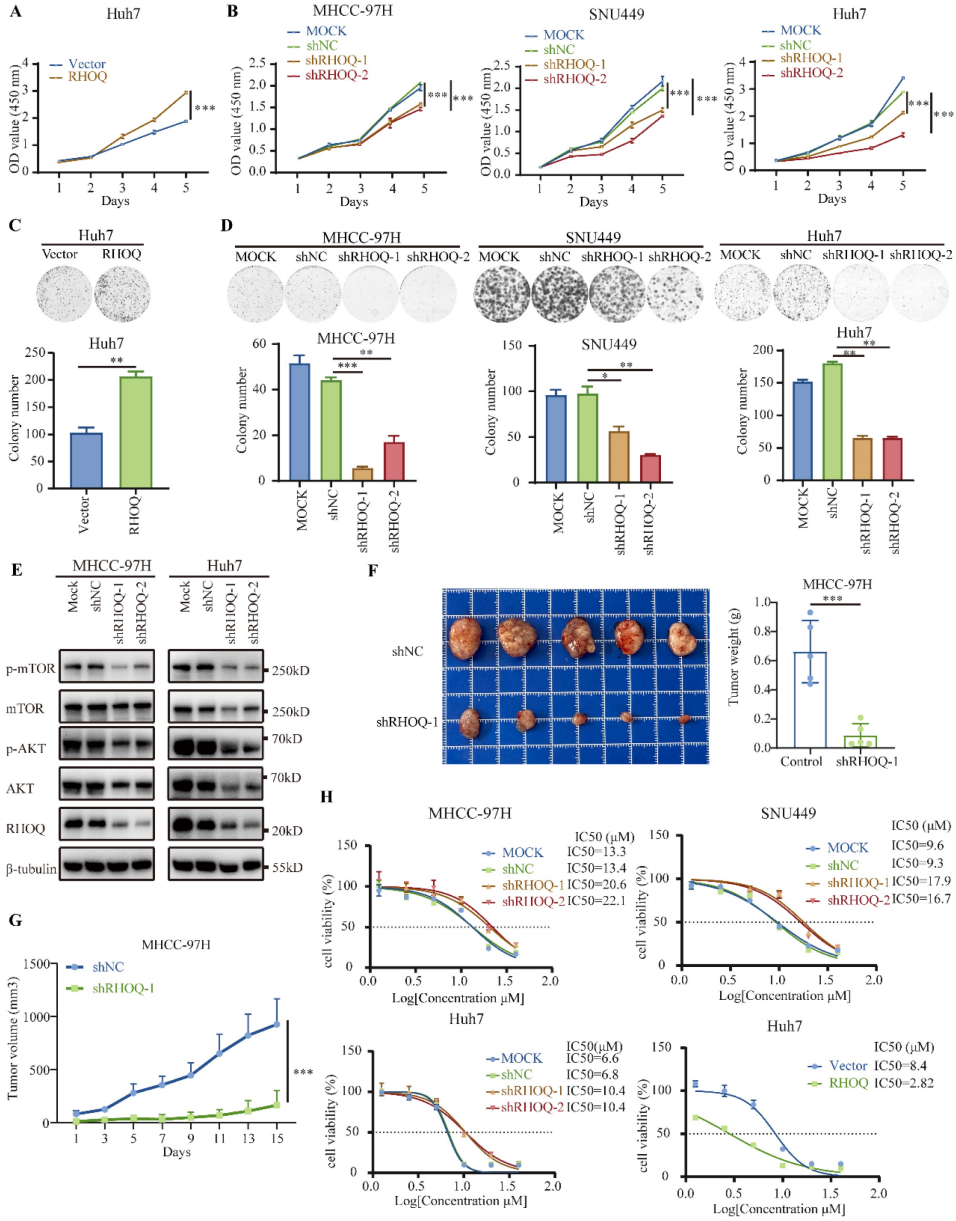
** RHOQ promotes HCC cells' proliferation and modulates sensitivity to nebivolol.** A, B, C, D. Proliferation effects of RHOQ overexpression or knockdown were analyzed using CCK-8 assay (A, B) and colony formation assays (C, D). E. Western blotting of protein expressions of mTOR, p-mTOR, AKT, and p-AKT in HCC cells with knockdown of RHOQ. F, G. The subcutaneous tumor weights (F) and volumes (G) of MHCC-97H (n = 5) with RHOQ knockdown or shNC control in nude mice. H. IC_50_ of nebivolol were determined in MHCC-97H, Huh7, and SNU449 cell lines following stable RHOQ knockdown. IC_50_ of nebivolol were determined in Huh7 following stable RHOQ overexpression. Data are presented as mean ± SD. * *P* < 0.05, ** *P* < 0.01, *** *P* < 0.001.

RHOQ (Ras Homolog Family Member Q), a member of the Rho GTPase family, encodes a small GTP-binding protein involved in regulating cytoskeletal reorganization, cell polarity, and vesicle transport in cells 29-32. Although RHOQ has been reported to exhibit dysregulated expression in multiple cancer types and play either oncogenic or tumor-suppressing roles 29, 33, its function in HCC remains unclear. Analysis of the DepMap cell line database revealed that RHOQ is essential for HCC cell proliferation, as supported by dependency scores (Fig. S7A). We detected the mRNA and protein expression levels of RHOQ in multiple HCC cell lines (Fig. S7B and C), and selected SNU449, MHCC-97H, and Huh7 cells for stable RHOQ knockdown (the target sequences of RHOQ were in Supplementary Table 3), along with Huh7 cells for stable RHOQ overexpression (Fig. S7D - G). CCK-8 assays demonstrated that RHOQ knockdown significantly suppressed the proliferation of Huh7, SNU449, and MHCC-97H cells, whereas RHOQ overexpression enhanced the proliferative capacity of Huh7 cells (Fig. 6A and B). Colony formation assay results further confirmed that RHOQ promoted the colony formation rate of HCC cells (Fig. 6C and D). Additionally, wound healing assays and Transwell migration and invasion assays indicated that RHOQ knockdown reduced the metastatic potential of Huh7, SNU449, and MHCC-97H cells, while RHOQ overexpression promoted migration and invasion in Huh7 cells (Fig. S8A - D).

To further investigate the molecular mechanisms by which RHOQ promotes HCC progression, we performed GSEA analysis based on transcriptomic data from 360 HCC samples in the TCGA cohort, stratified by the mean cutoff value of RHOQ expression. The results revealed significant enrichment of multiple signaling pathways in the high RHOQ expression group, including PI3K/AKT/mTOR, mitotic spindle, adherent junction, and focal adhesion (Fig. S9A). These findings are consistent with the previously observed phenotypes in which RHOQ enhances HCC proliferation and metastasis. The PI3K/AKT/mTOR signaling pathway is a crucial intracellular cascade that regulates fundamental cellular functions, including but not limited to cell growth, motility, survival, metabolism, and angiogenesis 34. The PI3K/AKT/mTOR axis is one of the most frequently altered pathways in human cancers 35. To further validate whether RHOQ influences HCC progression by regulating this pathway, we examined the activation status of key proteins in the PI3K/AKT/mTOR pathway. AKT, a key downstream effector of PI3K, and its phosphorylation (p-AKT), along with phosphorylation of mTOR (p-mTOR), are important markers of pathway activation. The results indicated that knockdown of RHOQ significantly suppressed the level of p-AKT and p-mTOR (Fig. 6E). These findings suggest that RHOQ promotes HCC progression by modulating the PI3K/AKT/mTOR signaling pathway.

To further evaluate the tumor-promoting effects of RHOQ *in vivo*, we established a subcutaneous xenograft model by implanting MHCC-97H cells with stable RHOQ knockdown or control. The results showed that RHOQ knockdown significantly inhibited tumor growth (Fig. 6F and G). Together, the results demonstrate that RHOQ promotes both proliferative and metastatic phenotypes of HCC cells* in vitro* and* in vivo*.

Given our above findings that nebivolol treatment downregulates RHOQ expression, we next examined whether RHOQ expression influences cellular response to nebivolol. Following stable overexpression or knockdown of RHOQ, HCC cells were treated with nebivolol for 48 hours. RHOQ overexpression significantly increased sensitivity to nebivolol, while RHOQ knockdown reduced it (Fig. 6H). Specifically, in Huh7 cells, RHOQ overexpression decreased IC_50_ values from 8.40 μM to 2.82 μM, reflecting a 66.4% increase in sensitivity (Fig. 6H). Conversely, RHOQ knockdown reduced sensitivity by 52.9% to 92.5% across MHCC-97H, Huh7, and SNU449 cell lines (Fig. 6H). These results suggest that nebivolol inhibits HCC progression, at least partially, through downregulation of RHOQ.

### The combination of nebivolol and lenvatinib exerts synergistic antitumor activity in HCC

**Figure 7:**
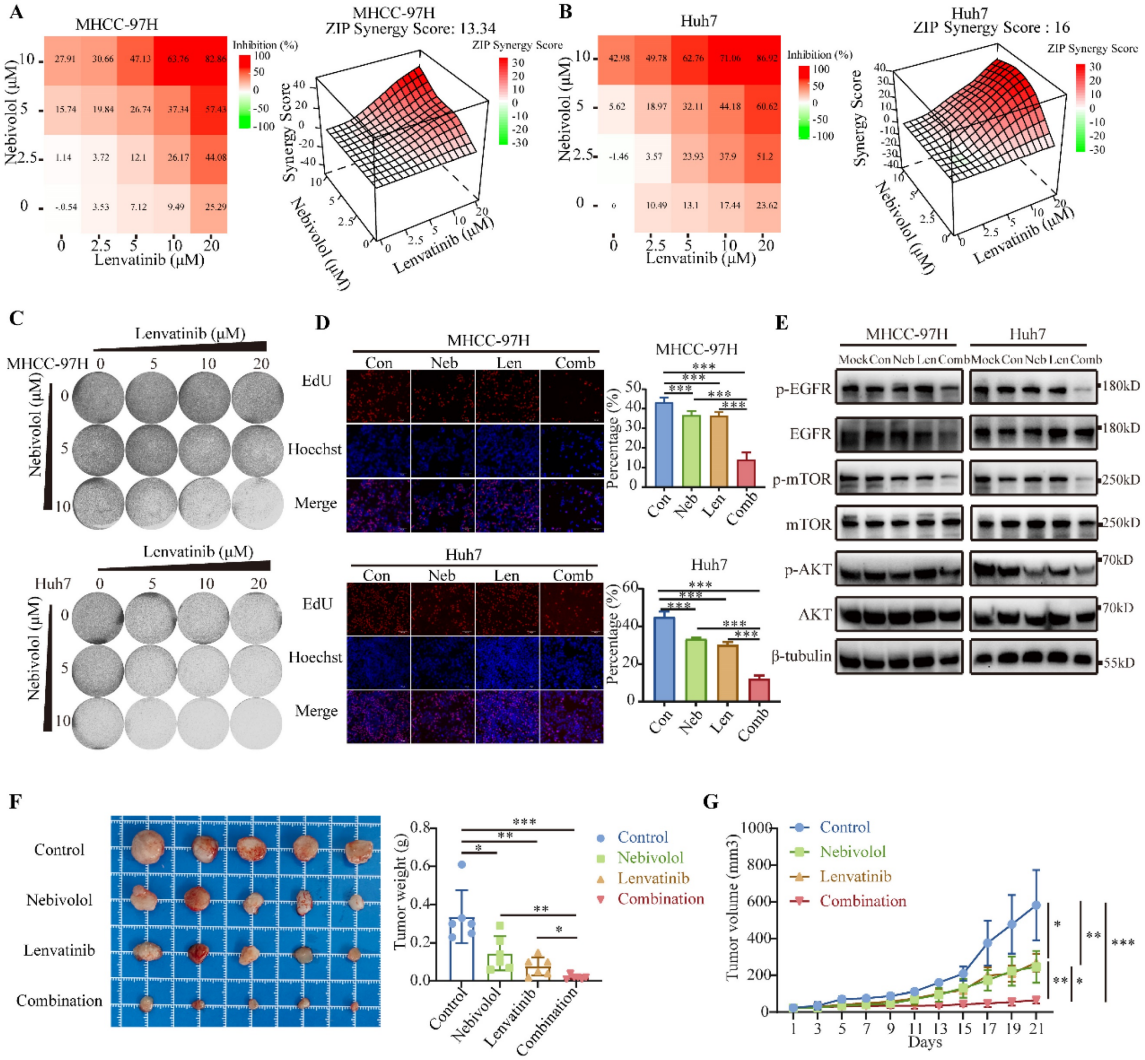
** The combination of nebivolol and lenvatinib exerts synergistic antitumor activity in HCC.** A, B. Heatmaps of drug combination response. Nebivolol and lenvatinib act synergistically on MHCC-97H and Huh7 cells. Cells were treated with the indicated concentrations of nebivolol and lenvatinib for 48 hours, ZIP Synergy Scores (synergy: >0; strong synergy: >10) were calculated using SynergyFinder software. C. Colony formation assays for the combination therapy of nebivolol and Lenvatinib. D. Evaluate cell proliferation following combination therapy with nebivolol and lenvatinib using the EdU assay. Scale bar = 100 μm. E. Western blotting of protein expressions of EGFR, p-EGFR, mTOR, p-mTOR, AKT, and p-AKT in HCC cells treated with lenvatinib (5 μM) alone or nebivolol (Huh7 4 μM, MHCC-97H 14 μM) alone or combination of two drugs. Con, Control. Neb, Nebivolol. Len, Lenvatinib. Comb, Combination. F, G. The subcutaneous tumor weights (F) and volumes (G) of MHCC-97H (n = 5) in nude mice treated with lenvatinib (4 mg/kg) alone or nebivolol (20 mg/kg) alone or combination of two drugs by peritoneal injection. Data are presented as mean ± SD. * *P* < 0.05, ** *P* < 0.01, *** *P* < 0.001.

Lenvatinib, a multi-targeted tyrosine kinase inhibitor (TKI), is a first-line treatment for advanced HCC. However, its long-term efficacy is often limited by primary and acquired resistance in a substantial proportion of patients 36. Studies have shown that EGFR is frequently overexpressed and aberrantly activated in HCC, contributing significantly to disease progression and treatment response 37-39. Notably, elevated EGFR signaling is closely associated with lenvatinib resistance in HCC cells, indicating that EGFR inhibition could be a promising strategy to overcome this resistance 39, 40. To explore the effect of nebivolol on HCC-associated signaling, we performed Western blot analysis to assess key molecules involved in tumor progression. The results revealed that nebivolol significantly reduced the phosphorylation of EGFR and its critical downstream effectors—AKT, and mTOR—in HCC cells (Fig. S10A). This demonstrates that nebivolol effectively suppresses the EGFR-driven pro-survival and proliferative signaling network. Hypertension has also been identified as one of the most common adverse events of any grade associated with lenvatinib therapy in multiple clinical studies 6, 41, 42. Nebivolol, an antihypertensive agent with potential antitumor properties, may therefore offer dual benefits when combined with Lenvatinib. We treated HCC cell lines with different concentrations of lenvatinib combined with nebivolol. CCK-8 assay revealed that the combination of the two drugs exhibited stronger antitumor activity than either agent alone. Using online SynergyFinder software, we calculated ZIP synergy score based on the concentration gradient and corresponding inhibition indices. The average ZIP synergy scores were 13.34, 12.90, and 16.00 in MHCC-97H, SNU449, and Huh7 cells, respectively, indicating pronounced synergistic effects (Fig. 7A, B, and Fig. S11A). Colony formation assays and EdU detection both indicated that nebivolol enhances the cytotoxic effects of lenvatinib (Fig. 7C, D, and Fig. S11B, C). We further examined the phosphorylation levels of EGFR and its downstream signaling molecules in HCC cells treated with nebivolol, lenvatinib monotherapy, and combination therapy. Results demonstrated that the combination of nebivolol and lenvatinib significantly inhibited the phosphorylation activation of EGFR and its downstream signaling molecules (Fig. 7E). Based on these findings, we hypothesize that nebivolol may exert synergistic anticancer effects with lenvatinib by suppressing the activation of EGFR-related signaling pathways.

We evaluated the synergistic effect of nebivolol and lenvatinib in an MHCC-97H xenograft nude mouse model. Tumor volume and weight were significantly reduced in the combination group compared to monotherapy groups (Fig. 7F, G). No significant differences were observed in body weight, liver-to-body weight ratio, or lung-to-body weight ratio among the experimental groups (Fig. S12A and B).

In summary, our findings demonstrate that the combination of nebivolol and lenvatinib enhances antitumor activity against HCC both* in vitro* and* in vivo*, supporting its potential as a novel therapeutic strategy.

## Discussion

Hepatocellular carcinoma remains one of the most common and lethal cancers worldwide, underscoring the urgent need for improving treatment strategies and developing new drugs. Growing evidence indicates that CVD and cancer are interrelated, and the cardiovascular toxicity caused by anticancer treatments increases the risk of CVD in cancer survivors 9, 10, 12. In this study, through screening of cardiovascular disease treatment drugs, we identified for the first time that the β_1_-adrenergic receptor antagonist nebivolol exhibits significant anti-HCC activity both *in vitro* and *in vivo*. This finding provides new directions for treatment options and mechanistic research in HCC.

Nebivolol is an FDA-approved β_1_-adrenergic receptor antagonist used for the treatment of hypertension 14. Beyond its cardiovascular applications, recent studies have revealed antitumor properties of nebivolol across multiple cancer types, including lung cancer, melanoma, colorectal cancer, breast cancer, neuroblastoma, and esophageal cancer 16-18, 43-45. Unlike many other beta-blockers, nebivolol has minimal effects on glucose and lipid metabolism, and evidence suggests it does not increase the risk of insulin resistance or dyslipidemia, rendering it suitable for hypertensive patients with diabetes or metabolic syndrome 46. In the present study, we demonstrated that nebivolol effectively suppresses HCC growth in both cellular and animal models. Consistent with previous reports in other cancers 21, we confirmed that nebivolol induces apoptosis and G0/G1 cell cycle arrest in HCC cell lines. Therefore, nebivolol may achieve synergistic antitumor effects through a dual mechanism: on one hand, it blocks the cell cycle to limit proliferation, and on the other hand, it triggers apoptosis to eliminate abnormal cells. This “dual-hit” strategy can effectively delay the development of drug resistance.

β-adrenergic signaling modulates multiple cellular processes and has been demonstrated to promote cancer initiation and progression. Studies indicate that β-blockers exhibit antitumor activity across various cancer models 18, 24, 27. However, our results indicate that the inhibitory effects of nebivolol on HCC cells are independent of β₁-adrenergic receptor blockade. RNA sequencing and subsequent experimental validation revealed that RHOQ serves as a key downstream target through which nebivolol exerts its anti-HCC activity. Although previous studies have shown that the β_1_ adrenergic receptors act as immune checkpoints modulated by the sympathetic nervous system and contribute to T cell exhaustion 25, we did not observe significant changes in T cell proportions following nebivolol treatment. Nonetheless, further investigation is warranted to determine whether nebivolol modulates other immune components within the tumor microenvironment.

RHOQ, a member of the Cdc42 subfamily of Rho GTPase family, participates in biological processes such as cell polarity establishment, vesicle transport, and cytoskeletal dynamic remodeling by regulating the GTP/GDP binding state transition 31, 32, 47, 48. Studies have shown that RHOQ acts as a regulatory factor to modulate invasive pseudopod extracellular vesicles, participating in the regulation of matrix degradation, invasion, and metastasis in breast cancer 29. Our functional studies demonstrated that RHOQ expression is closely associated with the malignant progression of hepatocellular carcinoma and poor patient prognosis. The PI3K/AKT/mTOR axis is one of the most frequently altered pathways in human cancers 35. We found that RHOQ promotes HCC progression by modulating the PI3K/AKT/mTOR signaling pathway. EGFR and RHO GTPases serve as two pivotal hubs in cellular signaling networks. Accumulating evidence indicates that these pathways are not functionally independent but are closely interconnected. As a crucial upstream regulator, EGFR can directly or indirectly activate RHO GTPases. For instance, ligand binding to EGFR activates GEFs such as VAV2, which in turn activate Rho GTPases. This activation induces actin cytoskeletal reorganization, which ultimately drives cell migration and invasion 49. Studies in breast cancer models have demonstrated that integrin-mediated cell adhesion promotes EGFR clustering and activation, thereby inducing Rho GTPase activation 50. Collectively, these findings indicate that EGFR signaling serves as a critical upstream regulatory node for RHO GTPase activity. Although we have not yet directly identified upstream factors regulating RHOQ expression through experiments, the series of results from this study indicate that nebivolol can effectively inhibit the activation of EGFR and its downstream key signaling molecules. Based on this, we propose a plausible scientific hypothesis: nebivolol may indirectly downregulate the expression or activity of RHOQ, a member of the Rho family, by inhibiting EGFR and its downstream signaling network.

Lenvatinib is a multi-kinase inhibitor and first-line drug used to treat patients with advanced HCC 51. Numerous studies have identified various factors associated with lenvatinib resistance, such as EGFR activation, m7G tRNA modification, serine metabolism reprogramming, and regulated cell death, which reduce the efficacy of lenvatinib in HCC 52-54. EGFR overactivation is associated with HCC cell resistance to lenvatinib 38, 39. In our study, we've proven that the combination of nebivolol and lenvatinib significantly inhibited the phosphorylation activation of EGFR and its downstream signaling molecules. Therefore, we evaluated the combination of nebivolol with lenvatinib.* In vitro* and *in vivo* experimental results clearly demonstrated that nebivolol enhances the anti-HCC effects of lenvatinib, providing direction and evidence for clinical trials and personalized treatment. It is worth noting that the efficacy of nebivolol in treating liver cancer requires further clinical trial research.

**Figure 8:**
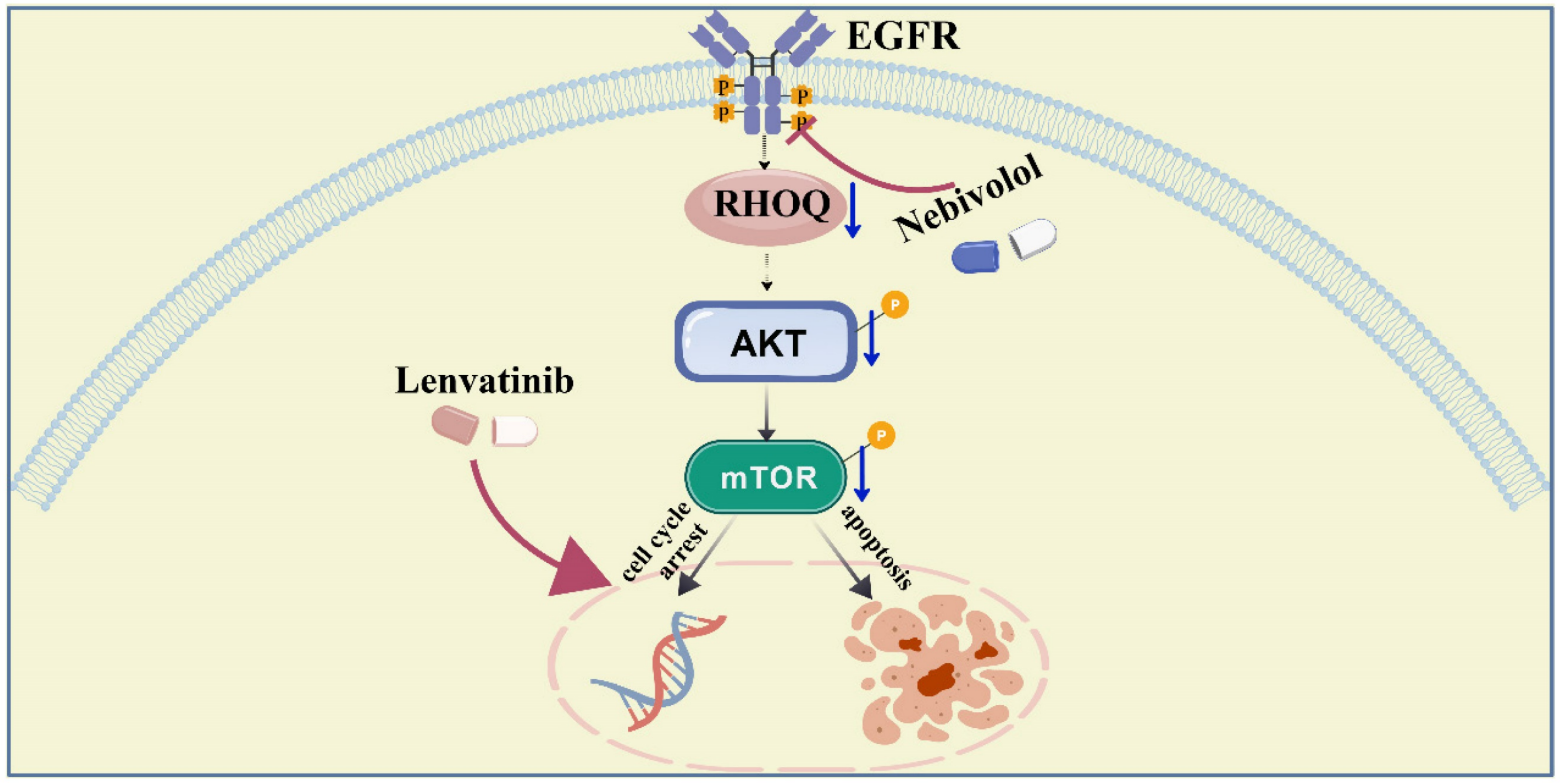
A schema showing that nebivolol inhibits hepatocellular carcinoma via RHOQ and enhances the efficacy of Lenvatinib. Created with BioGDP.com [55].

In summary, this study provides the first evidence of the antitumor effects of nebivolol in HCC. We demonstrate that nebivolol suppresses HCC through downregulation of RHOQ — a molecule overexpressed in HCC tissues and associated with poor prognosis. Furthermore, nebivolol synergizes with lenvatinib to enhance antitumor efficacy by suppressing the activation of EGFR-related signaling pathways (Fig. 8). These findings provide theoretical insights into the mechanism of action of nebivolol and support the exploration of RHOQ as a novel therapeutic target in HCC.

## Supplementary Material

Supplementary figures and tables.

## Data Availability

All data generated or analyzed during this study are included in this manuscript and its supplementary information files.

## Abbreviations

HCC: hepatocellular carcinoma
ATCC: American Type Culture Collection
FBS: fetal bovine serum
CCK-8: Cell Counting Kit-8
CVD: Cardiovascular disease
NSCLC: non-small cell lung cancer
DMSO: Dimethyl sulfoxide
DMEM: Dulbecco's Modified Eagle Medium
RPMI 1640: Roswell Park Memorial Institute 1640
PCR: Polymerase chain reaction
PBS: Phosphate buffer saline
IC_50_: Half-maximal inhibitory concentration
IHC: Immunohistochemistry
RHOQ: Ras Homolog Family Member Q
TKI: tyrosine kinase inhibitor
HR: Hazard Ratio
