# Supplementary material for: Nebivolol Inhibits Hepatocellular Carcinoma via RHOQ and Enhances the Efficacy of Lenvatinib
Source: Int J Biol Sci. 2026 Mar 25;22(7):3617–34. doi: 10.7150/ijbs.127395 (PMC13086015; doi:10.7150/ijbs.127395)
Supplement: Supplementary file 1 — Supplementary figures and tables. [file ijbsv22p3617s1.pdf]

Supplementary Figures and legends

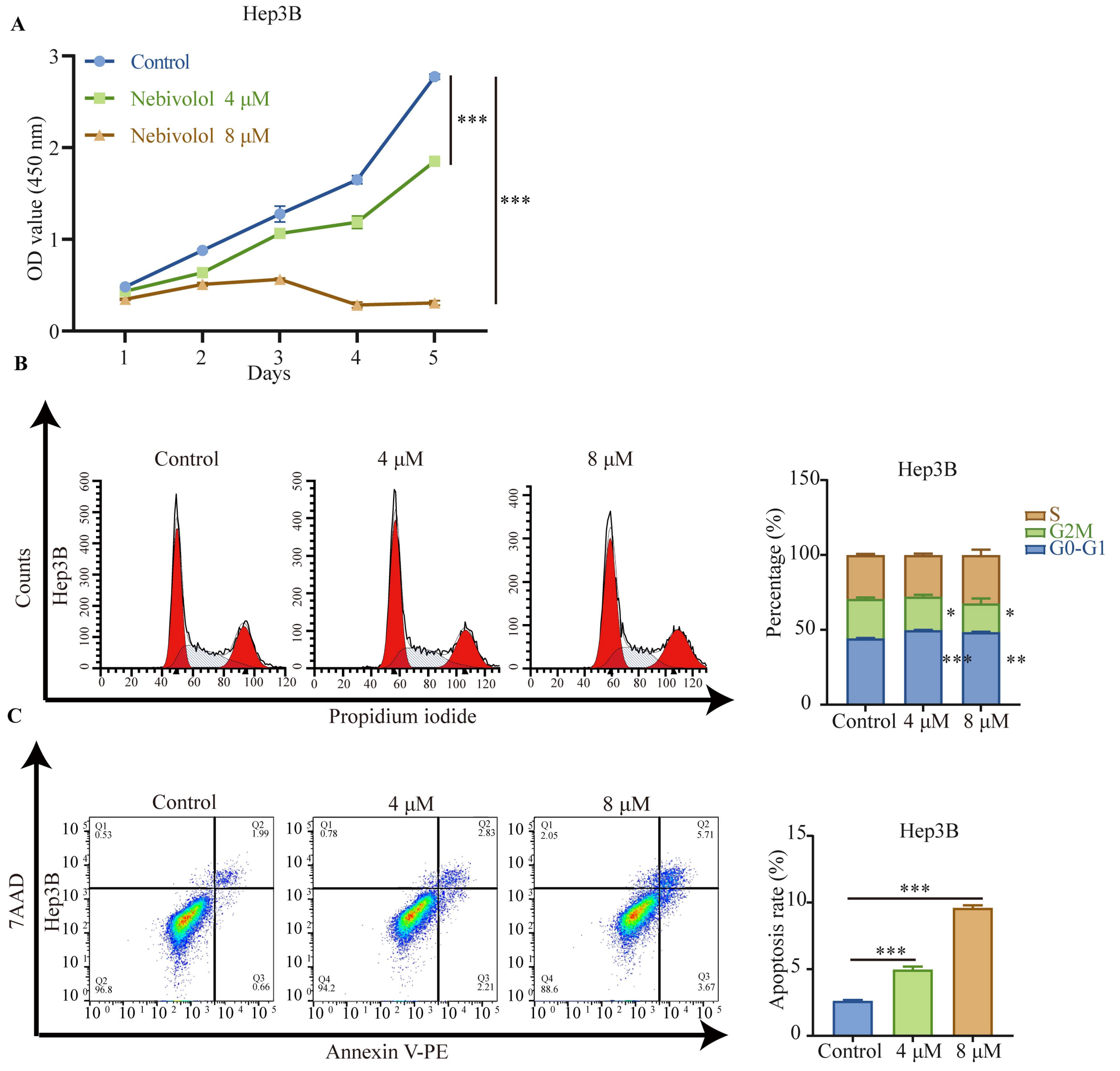

Figure S1. Nebivolol inhibits the proliferation of HCC cells *in vitro*.

A. Cell viability of Hep3B after 48 hours treatment with nebivolol. B. Representative images and quantification of Hep3B cell cycle distribution treated with different concentrations of nebivolol for 48 hours. C. Representative images and quantification of apoptotic Hep3B cells treated with different concentrations of nebivolol for 48 hours. Data are presented as mean ± SD. * $P < 0.05$, ** $P < 0.01$, *** $P < 0.001$.

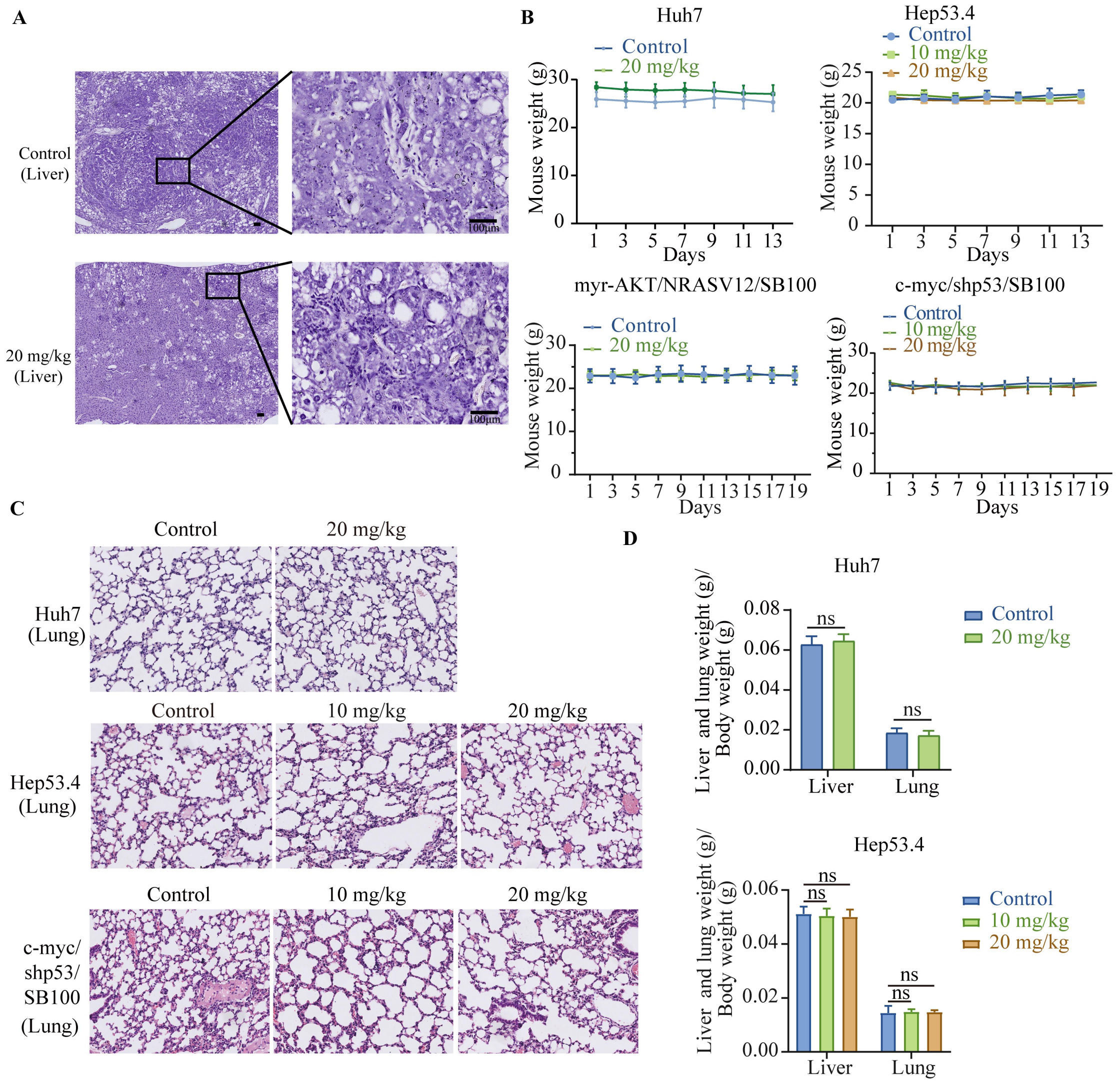

Figure S2. The toxic effects of nebivolol *in vivo*.

A. Representative images of H&E-stained liver tumors induced by myr-AKT/NRASV12/SB100 plasmids. Scale bar = 100 μm. B. Body weights of mice during control or nebivolol treatment. C. Representative images of H&E stained lungs of mice treated with control or nebivolol treatment. D. The liver/body weight and lung/body weight ratios of mice treated with nebivolol or control.

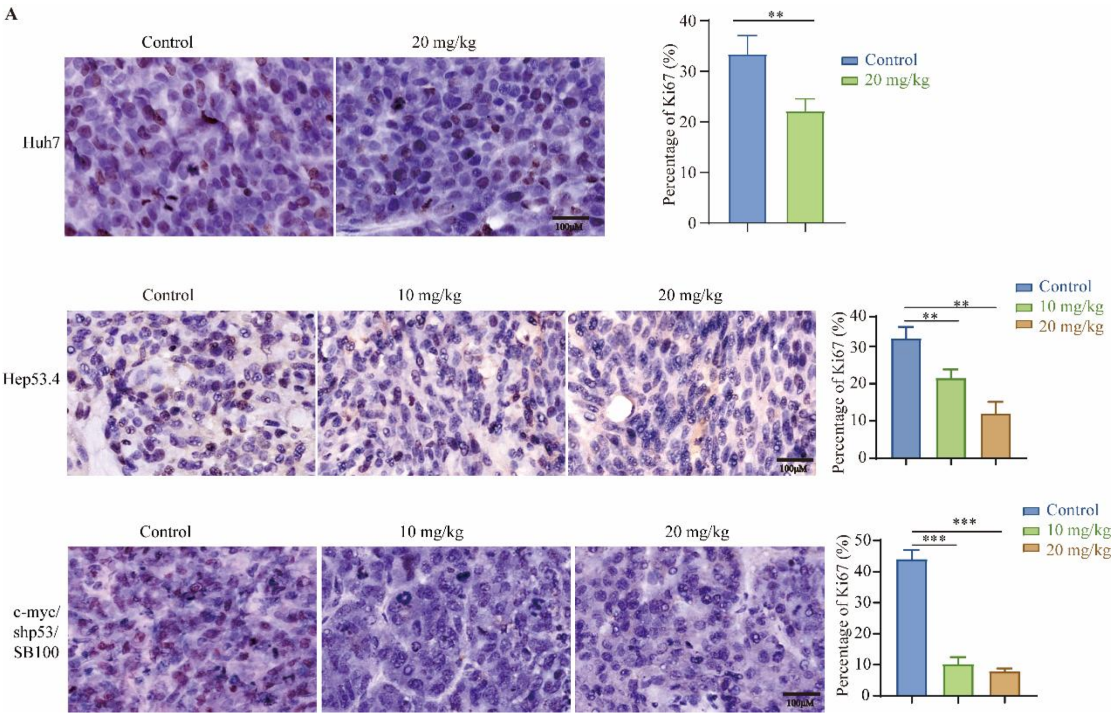

Figure S3. Nebivolol inhibits the proliferation of HCC cells *in vivo*.

A. The expression of Ki67 in tumor tissues treated with nebivolol was evaluated by IHC. Scale bar = 100 μm. Data are presented as mean ± SD. * $P < 0.05$, ** $P < 0.01$, *** $P < 0.001$.

A

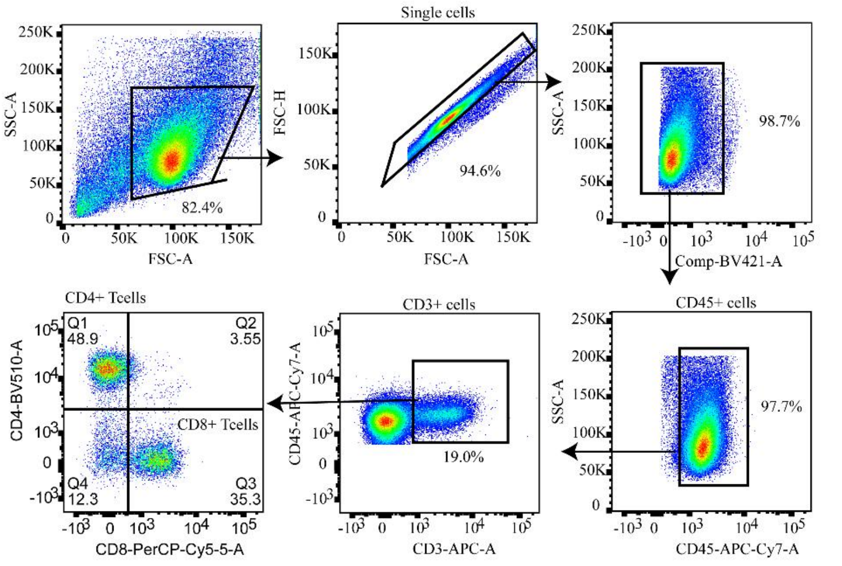

Figure S4. Gating strategy on samples for CD8+ T cells.

A. Gating strategy on spleen and tumor samples for CD8+ T cells.

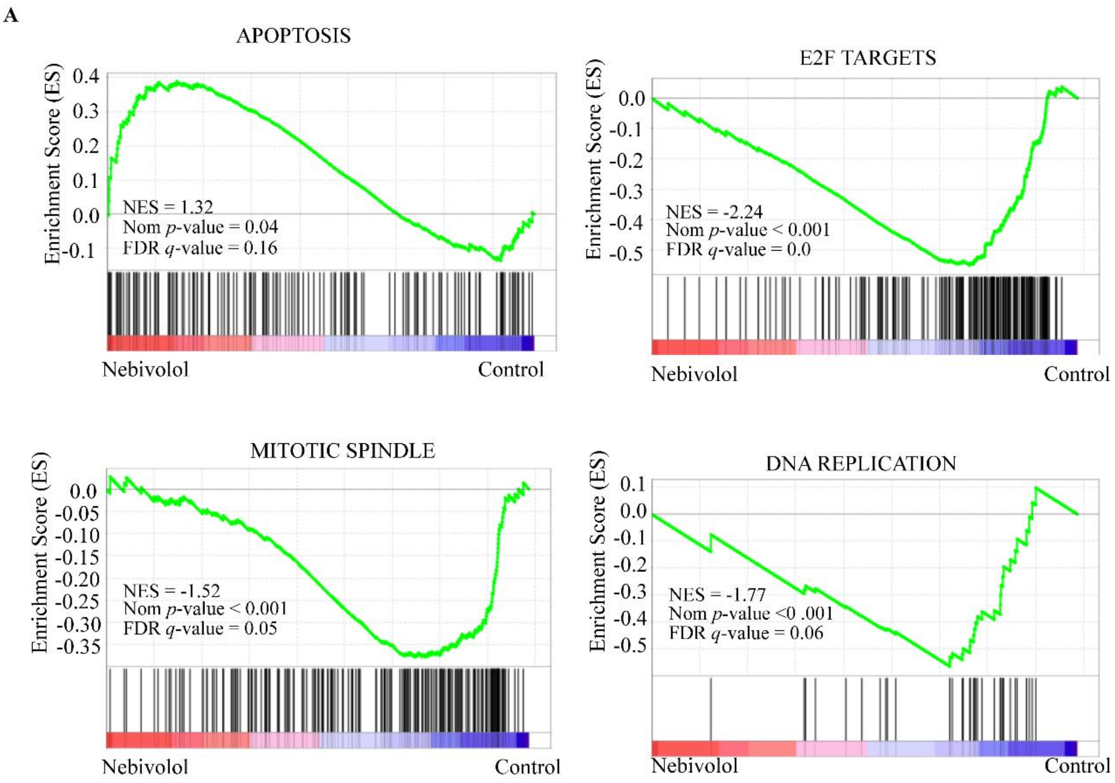

Figure S5. GSEA analysis of genes regulated by nebivolol.

A. GSEA analysis comparing transcriptomes of HCC cells treated with Nebivolol to control. NSE, normalized enrichment score.

A

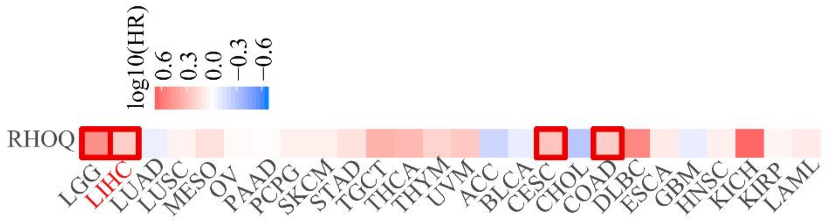

Figure S6. RHOQ predicts poor prognosis.

A. Association between RHOQ expression and patient survival in pan-cancer analysis.

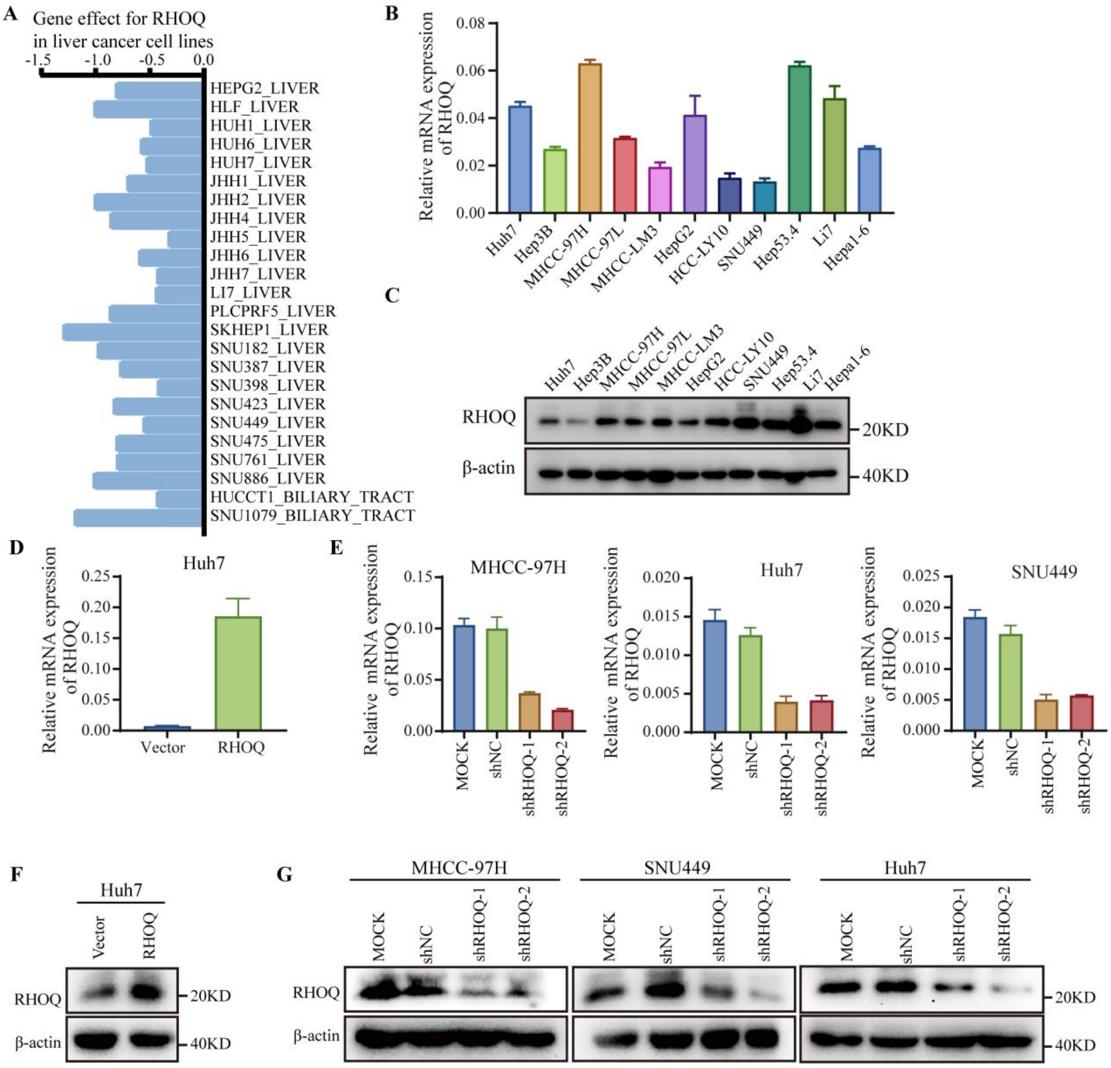

Figure S7. The mRNA and protein expressions of RHOQ in HCC cells.

A. Gene effect for RHOQ in liver cancer cell lines (0 indicates no dependence; the larger the negative value, the greater the dependence). B. Relative mRNA expression of RHOQ in different HCC cell lines. C. Protein expression levels of RHOQ in different HCC cell lines. D, E. Efficiencies of overexpression and knockdown of RHOQ verified by qRT-PCR. F, G. Efficiencies of overexpression and knockdown of RHOQ verified by western blotting.

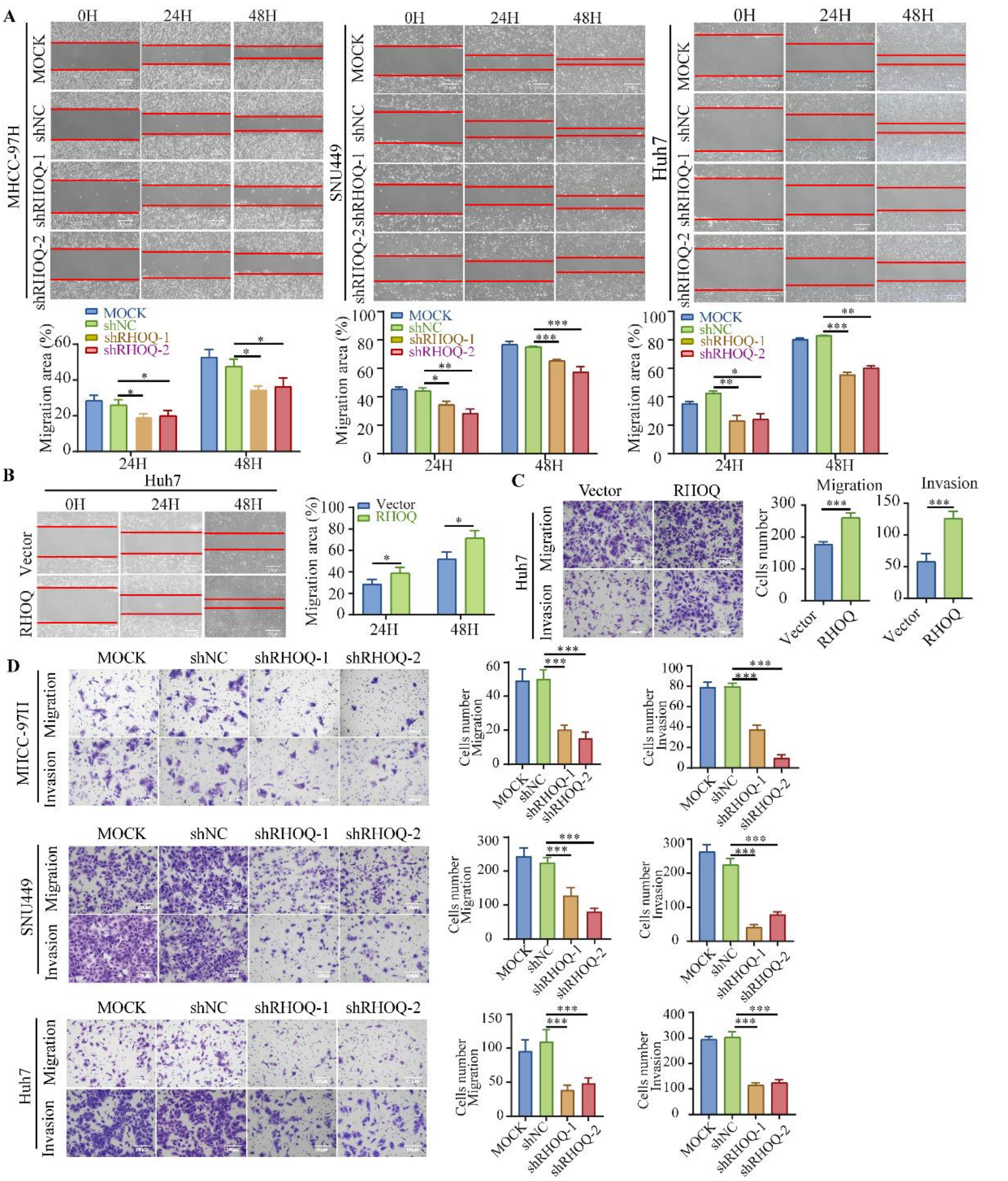

Figure S8. RHOQ promotes migration and invasion of HCC in vitro.

A, B. Wound healing images and quantification results of each group. C, D. Migration and invasion transwell images and quantification results of each group. Data are presented as mean ± SD. * $P < 0.05$, ** $P < 0.01$, *** $P < 0.001$.

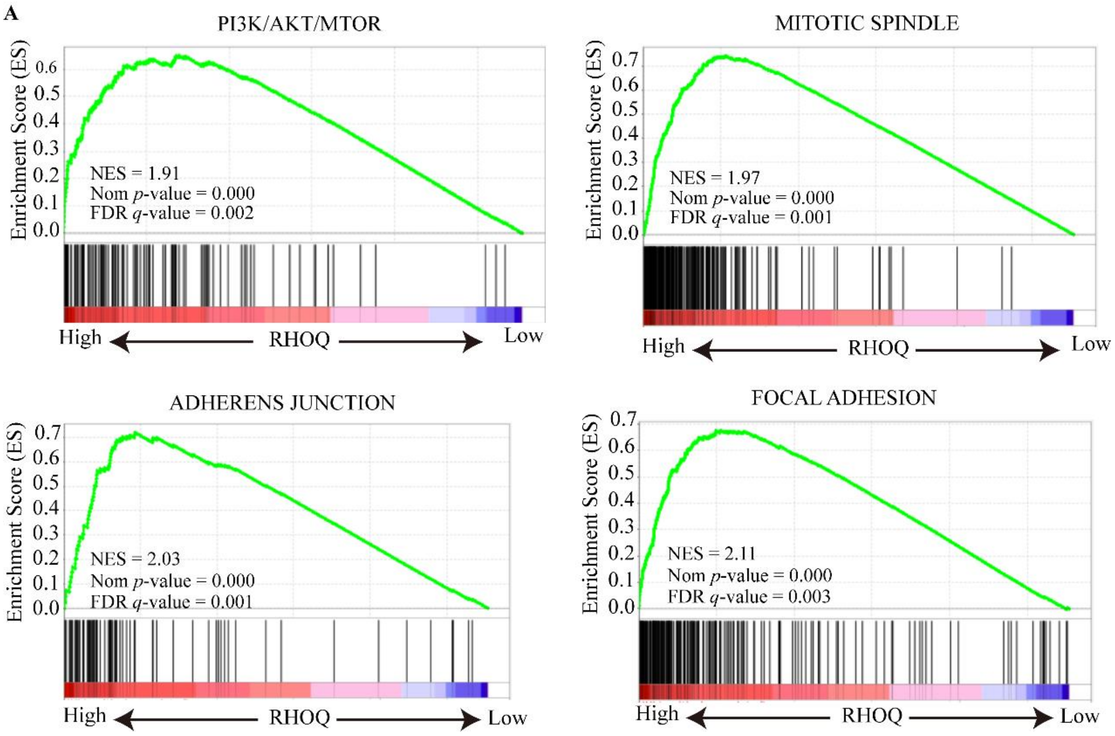

Figure S9. GSEA analysis of genes regulated by RHOQ.

A. GSEA analysis performed in a TCGA cohort of HCC tissues stratified by mean cut-off value of RHOQ expression. NSE, normalized enrichment score.

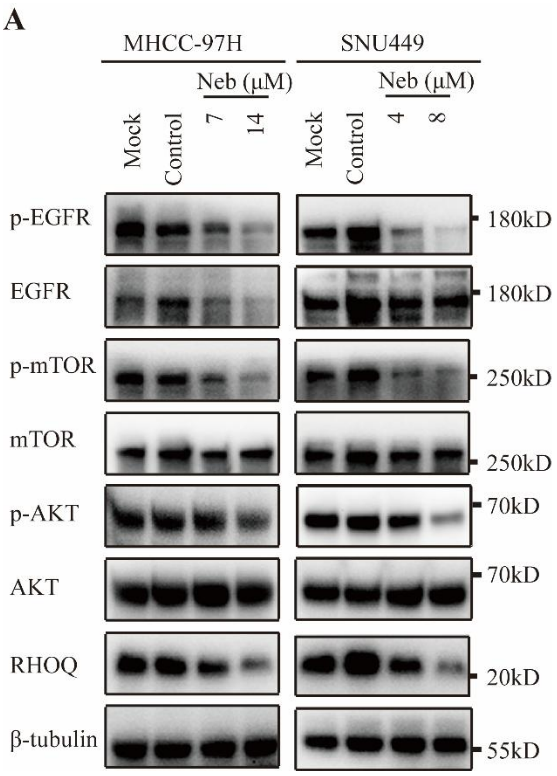

Figure S10. Nebivolol downregulates EGFR-related signaling pathways.

A. Western blotting of protein expressions of EGFR, p-EGFR, mTOR, p-mTOR, AKT, p-AKT, and RHOQ in HCC cells treated with control (DMSO) or nebivolol. Neb, nebivolol.

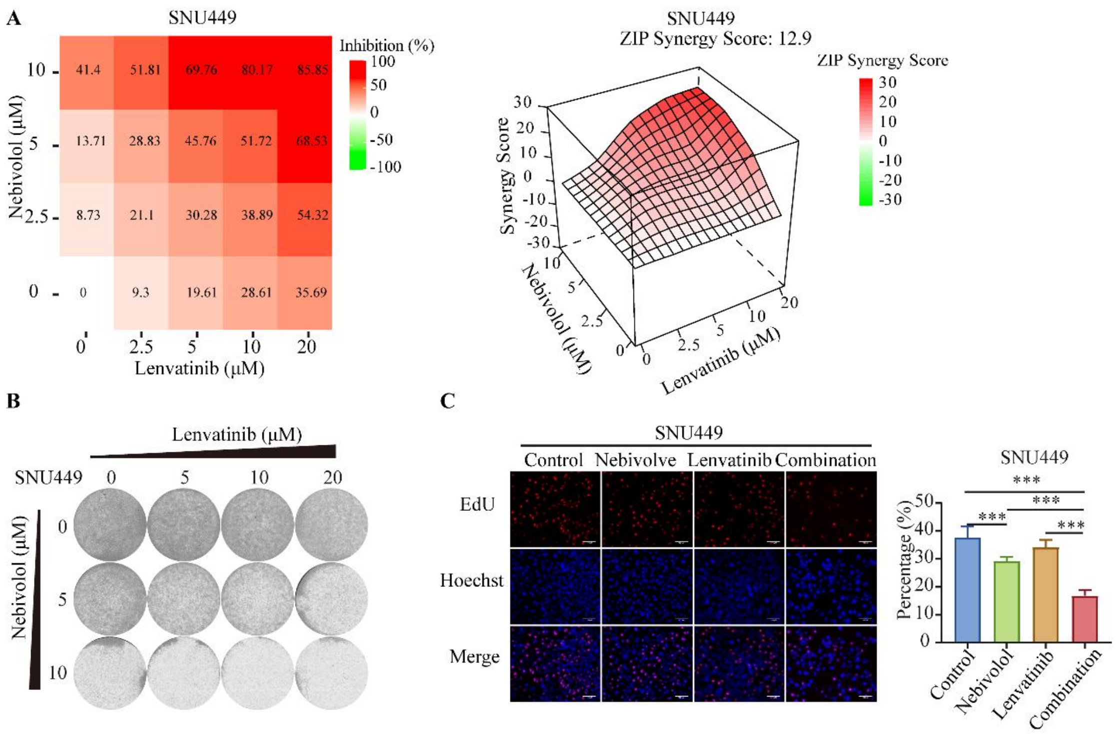

Figure S11. Nebivolol synergistically enhances the cytotoxicity of Lenvatinib against HCC cells.

A. Heatmaps of drug combination response. Nebivolol and lenvatinib act synergistically on SNU449 cells. B. Colony formation assay for the combination of nebivolol and lenvatinib. C. Cell proliferation following combination therapy with nebivolol and lenvatinib was assessed using the EdU assay. Data are presented as mean ± SD. * $P < 0.05$, ** $P < 0.01$, *** $P < 0.001$.

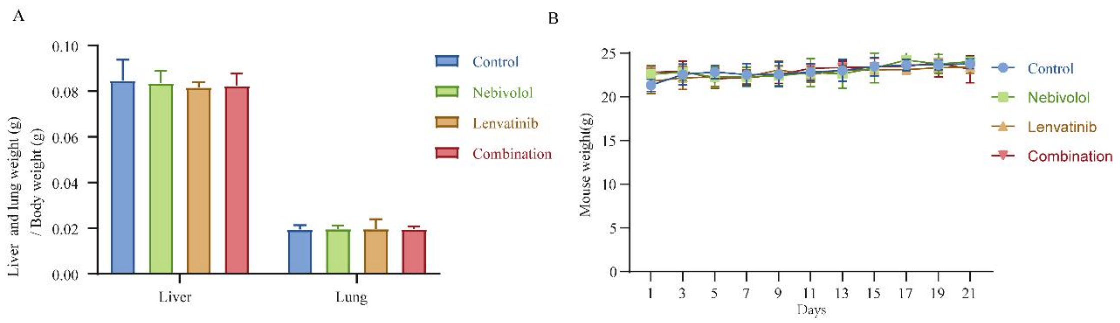

Figure S12. Nebivolol combined with lenvatinib showed synergic effect in HCC treatment.

A. The liver/body weight and lung/body weight ratios of mice treated with control or nebivolol alone or lenvatinib alone or combination of the two drugs. B. Body weights of mice during control or nebivolol alone or lenvatinib alone or combination of the two drugs treatment.

Supplementary Tables and legends

Table S1. Primer sequences of genes used in the study.

Primers	Primer Sequences
ADRA1A-F	TCATGCCCATTGGGACACAC
ADRA1A-R	CAGTGGTGGGTTTCATGCTC
ADRA1B-F	TCCCTCTGGCGGTCATTCTA
ADRA1B-R	TGGAGAACAAGGAGCCAAGC
ADRA1D-F	GCTACGCTGTCTTCTCCTCC
ADRA1D-R	CGGGAACAAGGAGCCGAG
ADRA2A-F	TCATCGGAGTGTTCGTGGTG
ADRA2A-R	TTGAAGAGCGTGCGTGGC
ADRA2B-F	TTCGTGCTGGCTGTGGTC
ADRA2B-R	GTCCTGGTTGAAGATGGTGTAG
ADRA2C-F	ACCTTTGTGCTGGCTGTG
ADRA2C-R	GGTTGAAGACCGTGTAGATGA
ADRAB1-F	TGCTACAACGACCCCAAGTG
ADRAB1-R	CAGGTACACGAAGGCCATGA
ADRAB2-F	TCTGCTGGCTGCCCTTCT
ADRAB2-R	TGTTGCCGTTGCTGGAGT
ADRAB3-F	CTTGGGTCTCATCATGGGCA
ADRAB3-R	CAAGAAGCCCCGTCGAG
RHOQ-F	AAAGAGGAGTGGGTACCGGA
RHOQ-R	GCAGCATGCTCCTATCTCTT

Table S2-1. Genes downregulated by nebivolol treatment in the study.

gene id	gene name	gene id	gene name
ENSG00000165029	ABCA1	ENSG00000135709	KIAA0513
ENSG00000006125	AP2B1	ENSG00000112984	KIF20A
ENSG00000171681	ATF7IP	ENSG00000149792	MRPL49
ENSG00000122786	CALD1	ENSG00000183444	OR7E38P
ENSG00000164305	CASP3	ENSG00000154124	OTULIN
ENSG00000070831	CDC42	ENSG00000011304	PTBP1
ENSG00000138092	CENPO	ENSG00000198218	QRICH1
ENSG00000108821	COL1A1	ENSG00000119396	RAB14
ENSG00000173402	DAG1	ENSG00000119729	RHOQ
ENSG00000186871	ERCC6L	ENSG00000236762	RPL19P16
ENSG00000164970	FAM219A	ENSG00000133318	RTN3
ENSG00000178974	FBXO34	ENSG00000097033	SH3GLB1
ENSG00000182963	GJC1	ENSG00000064652	SNX24
ENSG00000088256	GNA11	ENSG00000099994	SUSD2
ENSG00000164104	HMGB2	ENSG00000157600	TMEM164
ENSG00000224578	HNRNPA1L3	ENSG00000167460	TPM4
ENSG00000255154	HTD2	ENSG00000178252	WDR6
ENSG00000137496	IL18BP	ENSG00000185947	ZNF267
ENSG00000100441	KHNYN		

Table S2-2. Genes upregulated by nebivolol treatment in the study.

gene id	gene name	gene id	gene name
ENSG00000063322	MED29	ENSG00000197355	UAP1L1
ENSG00000175197	DDIT3	ENSG00000166340	TPP1
ENSG00000116717	GADD45A	ENSG00000052802	MSMO1
ENSG00000128965	CHAC1	ENSG00000149150	SLC43A1
ENSG00000081041	CXCL2	ENSG00000124198	ARFGEF2
ENSG00000234072	GTF3C2-AS2	ENSG00000104549	SQLE
ENSG00000197965	MPZL1	ENSG00000100889	PCK2
ENSG00000037749	MFAP3	ENSG00000110921	MVK
ENSG00000255526	NEDD8-MDP1	ENSG00000169242	EFNA1
ENSG00000101255	TRIB3	ENSG00000135404	CD63
ENSG00000120129	DUSP1	ENSG00000262246	CORO7
ENSG00000169429	CXCL8	ENSG00000164713	BRI3
ENSG00000113739	STC2	ENSG00000123240	OPTN
ENSG00000213699	SLC35F6	ENSG00000157911	PEX10
ENSG00000187688	TRPV2	ENSG00000186480	INSIG1
ENSG00000172840	PDP2	ENSG00000111674	ENO2
ENSG00000116761	CTH	ENSG00000167508	MVD
ENSG00000153714	LURAP1L	ENSG00000188211	NCR3LG1
ENSG00000143570	SLC39A1	ENSG00000184602	SNN
ENSG00000270194	GOLGA4-AS1	ENSG00000154803	FLCN
ENSG00000151012	SLC7A11	ENSG00000011021	CLCN6
ENSG00000131979	GCH1	ENSG00000171365	CLCN5

ENSG00000140961	OSGIN1	ENSG00000167994	RAB3IL1
ENSG00000025039	RRAGD	ENSG00000180035	ZNF48
ENSG00000108828	VAT1	ENSG00000176978	DPP7
ENSG00000147872	PLIN2	ENSG00000184371	CSF1
ENSG00000134107	BHLHE40	ENSG00000157870	PRXL2B
ENSG00000041353	RAB27B	ENSG00000237264	FTH1P11
ENSG00000188760	TMEM198	ENSG00000070540	WIPI1
ENSG00000103249	CLCN7	ENSG00000171813	PWWP2B
ENSG00000087076	HSD17B14	ENSG00000119655	NPC2
ENSG00000161011	SQSTM1	ENSG00000164171	ITGA2
ENSG00000168209	DDIT4	ENSG00000118960	HS1BP3
ENSG00000128165	ADM2	ENSG00000008517	IL32
ENSG00000114796	KLHL24	ENSG00000119899	SLC17A5
ENSG00000070669	ASNS	ENSG00000160285	LSS
ENSG00000130513	GDF15	ENSG00000147164	SNX12
ENSG00000164251	F2RL1	ENSG00000167996	FTH1
ENSG00000166900	STX3	ENSG00000122912	SLC25A16
ENSG00000179598	PLD6	ENSG00000241095	CYP51A1P1
ENSG00000116954	RRAGC	ENSG00000172059	KLF11
ENSG00000134070	IRAK2	ENSG00000133134	BEX2
ENSG00000040608	RTN4R	ENSG00000130311	DDA1
ENSG00000134049	IER3IP1	ENSG00000138166	DUSP5
ENSG00000090339	ICAM1	ENSG00000177606	JUN
ENSG00000112972	HMGCS1	ENSG00000123836	PFKFB2

ENSG00000175938	ORAI3	ENSG00000106799	TGFBR1

Table S3. Target sequences of shRNA used in the study.

Target gene name	Target Sequences
shRHOQ-1	GCAAGACTGAATGATATGAAA
shRHOQ-2	CGGTGGTAAATCCAGCCTCAT
